# Supplementary material for: Effectiveness of adding inspiratory muscle training to a cardiac rehabilitation program in people with acute myocardial infarction revascularized by percutaneous coronary intervention (CARDIOINSPIRE): Study protocol for a randomized controlled trial
Source: PLoS One. 2026 Mar 10;21(3):e0343947. doi: 10.1371/journal.pone.0343947 (PMC12974859; doi:10.1371/journal.pone.0343947)
Supplement: S2 File — (PDF) [file pone.0343947.s002.pdf]

Proyecto CARDIOINSPIRE

“Efectividad de la adición del entrenamiento de la musculatura inspiratoria a un programa de rehabilitación cardiaca de personas con cardiopatía isquémica revascularizadas mediante angioplastia coronaria transluminal percutánea.”

Investigador Principal y doctorando:
José María Zuazagoitia De la Lama-Noriega

Centro donde se va a realizar:
Hospital Universitario Virgen de la Victoria de Málaga

Servicio donde se va a realizar:
Rehabilitación

Responsable asistencial en el Hospital Virgen de la Victoria
y co-directora de Tesis:
Dra. Adela María Gómez González

Co-director de Tesis;
Dr. José Antonio Moral Muñoz

1.- Resumen

La cardiopatía isquémica (CI) o enfermedad de las arterias coronarias consiste en el estrechamiento de la luz interna de las arterias que perfunden el corazón. A medida que disminuye la sección de la arteria, también lo hace el flujo de sangre y por tanto el aporte de oxígeno al miocardio, pudiendo provocar una angina de pecho o incluso un infarto agudo de miocardio. Es la principal causa de muerte por enfermedad cardiovascular, responsable de 9,44 millones de muertes en 2021 y 185 millones de años de vida ajustados en función de la discapacidad. Por esta razón es uno de los problemas de salud pública más importantes en todos los países del mundo que conlleva un alto gasto sanitario y social.

La estrategia no farmacológica con más evidencia en la actualidad para mejorar la calidad de vida de los pacientes y además prevenir ulteriores eventos cardiovasculares son los programas de rehabilitación cardiaca (PRC). Estos incluyen el abandono del tabaco, el control del resto de factores de riesgo cardiovascular, educación sanitaria, terapia psicológica y un programa de ejercicio multimodal. Se realizan dos modalidades de ejercicio, el cardiovascular o resistencia aérobica y el de fuerza. Existe evidencia limitada sobre la adición de un entrenamiento específico de la musculatura inspiratoria (EEMI) en las personas con CI. Por esto no se recomienda de forma rutinaria en las guías de práctica clínica. Por tanto, el presente proyecto de tesis, basado en un ensayo clínico, trata de aumentar el conocimiento sobre este tema.

En vista de lo anterior, el objetivo principal de este proyecto es analizar la efectividad de la adición del entrenamiento de la musculatura inspiratoria a un PRC de personas con cardiopatía isquémica revascularizadas mediante angioplastia coronaria transluminal percutánea (ACTP) tras 16 sesiones de intervención, en base a la capacidad funcional, además de la fuerza muscular, soporte social, ansiedad, depresión, afrontamiento de la enfermedad, disfunción sexual, calidad de vida, calidad del sueño, hábitos alimenticios y composición corporal. Por otra parte, los objetivos secundarios son conocer el perfil biopsicosocial de esta población y analizar las diferencias de género a través de un análisis de subgrupo.

Para alcanzar estos objetivos se realizará un ensayo clínico de bajo riesgo aleatorizado y controlado en paralelo en el Hospital Universitario Virgen de la Victoria de Málaga (HUVV). Se reclutará a personas de esta área sanitaria diagnosticadas de CI a las que se les haya realizado una ACTP. El grupo control realizará el programa de rehabilitación cardiaca habitual de 8 semanas de duración con 2 sesiones semanales de ejercicio multicomponente (cardiovascular y de fuerza), una sesión semanal de educación sanitaria y otra de terapia psicológica grupal. El grupo de intervención hará el mismo programa al que se le añadirá un EEMI. Se realizará con cargas del 70% del valor de la Presión Inspiratoria Máxima (PIM) completando 3 series de 10 repeticiones, 4 días a la semana, con 3 minutos de descanso entre series, durante las 8 semanas que dura el PRC. Se realizarán distintas mediciones y se pasarán diversas pruebas y cuestionarios antes y después del PRC y se analizará el efecto de la adición o no del EEMI sobre las variables a analizar mediante métodos estadísticos.

Se espera que algunas de las variables mejoren ya que hay cierta evidencia (baja y moderada) de ello, tal y como concluye una reciente revisión. Además, en poblaciones clínicas similares como pacientes con insuficiencia cardiaca (IC), si hay alto nivel de evidencia de que varios de estos parámetros mejoran. Por lo que se puede esperar que los resultados sean parecidos en CI. En aquellas variables sin evidencia previa, hipotetizamos que habrá una mejora, ya que el incremento de la PIM de forma aislada ha demostrado, en distintas poblaciones clínicas, mejorar la capacidad funcional y la calidad de vida de los sujetos de estudio.

Si finalmente se observan los beneficios esperados se conseguirá aumentar la evidencia actual sobre el uso del EEMI en los PRC para personas con CI y ACTP. En previsión de la evidencia acumulada de la efectividad del tratamiento propuesto, los resultados derivados del presente estudio se podrán recomendar incluir el EEMI como un componente fundamental más de los PRC para este subgrupo de pacientes.

Palabras clave: cardiopatía isquémica, rehabilitación cardiaca, entrenamiento de la musculatura inspiratoria.

2.- Antecedentes y estado actual del tema

2.1 La cardiopatía isquémica

Según la Fundación Española del Corazón[1], la cardiopatía isquémica (CI) es la enfermedad ocasionada por la arterosclerosis de las arterias coronarias, encargadas de proporcionar sangre al músculo cardiaco. La arterosclerosis coronaria es un proceso lento de formación de colágeno y acumulación de lípidos (grasas) y células inflamatorias (linfocitos) que va provocando el estrechamiento de la luz de las arterias coronarias[2]. Este proceso empieza en las primeras décadas de la vida, pero no presenta síntomas hasta que la estenosis de la arteria coronaria se hace tan grave que causa un desequilibrio entre el aporte de oxígeno al miocardio y sus necesidades metabólicas[3]. En este caso, se produce una isquemia miocárdica que puede ser crónica, conocida como síndrome coronario crónico o angina de pecho estable, o una oclusión súbita por trombosis de la arteria, lo que provoca una falta de oxigenación del miocardio que da lugar al síndrome coronario agudo también llamado angina inestable e infarto agudo de miocardio. La CI reduce la producción de fuerza, provoca arritmias y causa daño muscular, lo que provoca insuficiencia contráctil isquémica aguda del corazón[4]. Los pacientes con cardiopatía isquémica crónica tienen valoraciones más bajas de su vida social y de su afrontamiento de la rutina diaria[5].

La consecuencia de los procesos descritos anteriormente es que las personas que sufren CI van disminuyendo su tolerancia al ejercicio[6], funcionalidad[7] y rendimiento en las actividades de la vida diaria lo que les conduce a comportamientos sedentarios, empeorando su calidad de vida[8]. Además, se va estableciendo un círculo vicioso de inactividad y deterioro clínico y funcional que produce un empeoramiento general de la condición física y la atrofia de los músculos periféricos y respiratorios[9]. Todas estas circunstancias conducen a una disminución de la capacidad funcional y por tanto a un peor pronóstico de la enfermedad.

Con respecto a los factores de riesgo que derivan en el desarrollo de CI, existe una combinación de factores de riesgo socioeconómicos, metabólicos, conductuales y ambientales. Entre ellos se encuentra la edad avanzada, la hipertensión arterial, una dieta poco saludable, colesterol alto, diabetes, obesidad, tabaquismo, sedentarismo, estrés, antecedentes familiares y consumo nocivo de tóxicos entre otros. La mayor parte de estos factores son modificables y es uno de los pilares fundamentales de su tratamiento el conseguir que las personas afectadas cambien su estilo de vida[10,11]. La incidencia es mayor en los hombres ya que las mujeres se benefician de un efecto protector hormonal durante su vida fértil. Esta circunstancia desaparece con la menopausia lo que iguala los casos en ambos sexos a partir de los 45-50 años[12].

2.2 Epidemiología

Se estima que en el año 2020 vivían en todo el mundo 244.1 millones de personas con CI. De ellas, 141 millones eran hombres y 103.1 millones mujeres. Las regiones del Norte de África, Oriente Medio, Asia Central, Sur de Asia y Europa Oriental tuvieron las mayores tasas de prevalencia. La tasa de mortalidad mundial fue de 112.37 por cada 100.000 siendo esta también mayor en las regiones con más prevalencia[13].

La CI afecta aproximadamente a 126 millones de personas en todo el mundo, lo que representa el 1.72% de la población mundial. Es la principal causa de muerte, con 9 millones de muertes anuales. La incidencia comienza a aumentar en la cuarta década de vida y es mayor en hombres que en mujeres[14].
. En los países desarrollados, alrededor de un tercio de todas las muertes en personas mayores de 35 años se atribuyen a esta enfermedad. En Estados Unidos, se estima que casi la mitad de los hombres y aproximadamente un tercio de las mujeres experimentarán algún síntoma de CI durante su vida. En la Unión Europea, las enfermedades cardiovasculares representan el 40% de todas las muertes. Además, los costes económicos asociados a la CI son significativos. El tratamiento de las enfermedades cardiovasculares constituye el 54% del gasto total en salud, y contribuye aproximadamente a una cuarta parte de las pérdidas de productividad[15].

La enfermedad coronaria está estrechamente relacionada con el envejecimiento. Por lo tanto, a pesar de la disminución en su incidencia observada en las últimas décadas, todos los indicadores sugieren que su incidencia aumentará en los años venideros. Se espera que los síndromes coronarios aumenten entre un 69% y un 119% para el año 2049, principalmente debido al crecimiento de la población de pacientes mayores de 75 años. Para el grupo de pacientes entre 25 y 75 años, se proyecta que la incidencia se mantenga estable en las próximas décadas[16].

2.3 Clasificación

La CI puede manifestarse como[1]:

- **Infarto agudo de miocardio:** es la obstrucción aguda de una arteria coronaria. La consecuencia final es la muerte (necrosis) del territorio que irriga la arteria obstruida. La gravedad del infarto dependerá de la cantidad de músculo cardiaco que se pierda. Suele ser un evento inesperado que se puede presentar en personas sanas, aunque generalmente es más frecuente en quienes tienen factores de riesgo y en enfermos que ya han padecido otra manifestación de cardiopatía isquémica. La necrosis del territorio que se queda sin riego sanguíneo es progresiva. El daño se incrementa con el tiempo y, una vez muere la porción de músculo cardiaco, es imposible recuperar su función. El daño sí se puede interrumpir si el miocardio vuelve a recibir sangre mediante procedimientos que restituyan el flujo sanguíneo. En algunas ocasiones se puede producir una muerte súbita. El infarto de miocardio es la principal causa de muerte de hombres y mujeres en todo el mundo.

- **Angina de pecho estable (arteriopatía coronaria crónica):** se produce cuando se realiza un esfuerzo que requiere un mayor aporte de oxígeno al corazón y se normaliza cuando cesa el ejercicio, por lo que es una situación transitoria. En ese momento puede aparecer sintomatología y pueden registrarse cambios en el electrocardiograma.

- **Angina de pecho inestable:** incluye todas las formas de angina que se apartan del patrón típico de angina estable: angina de reposo, angina de inicio o angina progresiva. El desarrollo es incierto, pero no necesariamente negativo.

2.4 Tratamiento

Habitualmente consiste en la trombólisis (revascularización de la arteria o arterias cuyo flujo está comprometido) y puede hacerse de dos formas:

- Mediante cirugía abierta realizando un puente entre dos vasos que redirija la sangre hasta el miocardio afectado.
- Realizando una angioplastia coronaria transluminal percutánea (ACTP). Esta opción es menos invasiva y se utiliza un globo en la punta de un tubo delgado, llamado catéter, para ensanchar la arteria. Para mantener la arteria abierta normalmente se coloca un pequeño muelle.

2.5 Prevención secundaria

Una vez se ha producido el primer evento cardiovascular es importante evitar que vuelva a suceder. Es lo que se denomina prevención secundaria. En este sentido, la estrategia no farmacológica con más evidencia en la actualidad para reducir la mortalidad, discapacidad cardiovascular, mejorar la calidad de vida de los pacientes y además prevenir ulteriores eventos cardiovasculares, son los programas de rehabilitación cardiaca (PRC)[17]. Además, es conocida su costo-efectividad[18,19]. Estos incluyen el abandono del tabaco, el control del resto de factores de riesgo cardiovascular, educación sanitaria, terapia psicológica y un programa de ejercicio multimodal (entrenamiento cardiovascular y de fuerza)[20]. Se realizan habitualmente 2-3 sesiones de entrenamiento semanal durante 8-10 semanas. Mas adelante, en el apartado de intervención, se detalla el contenido del PRC.

2.6 Entrenamiento de la musculatura Inspiratoria

El entrenamiento específico de la musculatura inspiratoria (EEMI) consiste en la aplicación de cargas de trabajo, mayores a las habituales, a los músculos responsables de generar la presión negativa necesaria dentro del tórax para que se produzca la entrada de aire a los pulmones. Esta sobrecarga induce adaptaciones que producen un aumento de la fuerza de esta musculatura. Su utilización está muy extendida como parte del tratamiento de personas con enfermedades respiratorias crónicas y personas con insuficiencia cardiaca (IC). Es una intervención muy segura que disminuye la disnea y mejora la capacidad funcional y la calidad de vida en estos pacientes.[21–24].

La sobrecarga se consigue al inspirar a través de dispositivos específicos que contienen una válvula cuyo umbral puede regularse. Para decidir la presión a la que va a entrenar cada individuo hay que medir previamente su Presión

Inspiratoria Máxima (PIM). Los protocolos de entrenamiento a cargas altas, de entre el 50% y el 80% de la PIM, son los que obtienen los mejores resultados según la evidencia disponible para pacientes con IC y para pacientes revascularizados con injerto mediante cirugía abierta[10–12]. Por esto, se realizará la intervención con cargas del 70% del valor de la PIM completando 3 series de 10 repeticiones, 4 días a la semana, con 3 minutos de descanso entre series, durante las 8 semanas que dura el PRC.

3.- Hipótesis y Objetivos

3.1 Justificación

En vista de la perspectiva presentada anteriormente y de la literatura disponible, resulta pertinente llevar a cabo un ensayo clínico aleatorio de bajo riesgo en el que se evalúe la efectividad de la adición del EEMI a un PRC de personas con CI con ACTP. Una revisión publicada recientemente[9], concluye que la calidad de la evidencia del entrenamiento de la musculatura inspiratoria para la mejora del consumo máximo de oxígeno y la fuerza muscular espiratoria en esta población es muy baja. No obstante, hay que tener en cuenta que la mayoría de los ensayos clínicos llevados a cabo hasta la fecha se han realizado sobre pacientes intervenidos mediante cirugía abierta[9], por lo que la literatura es escasa en personas intervenidas percutáneamente. En este sentido, los resultados de nuestro estudio estarán específicamente enfocados en los pacientes revascularizados percutáneamente, con lo que aumentará el conocimiento de los efectos de esta intervención en el subgrupo de pacientes con CI y ACTP sobre estas y otras variables. Aunque los PRC han demostrado ser efectivos[17,25], existe evidencia limitada sobre la adición de un EEMI en pacientes con CI. Las guías de práctica clínica no recomiendan rutinariamente el EEMI debido a esta falta de evidencia, por lo que se pretende arrojar luz sobre su efectividad.

3.2 Hipótesis

H1.- La adición del entrenamiento de la musculatura inspiratoria a un programa de rehabilitación cardiaca de personas con cardiopatía isquémica revascularizadas percutáneamente provocará mejores resultados a nivel de capacidad funcional y de fuerza muscular, además del soporte social, ansiedad, depresión, afrontamiento de la enfermedad, disfunción sexual, calidad de vida, calidad del sueño, hábitos alimenticios y composición corporal, tras 16 sesiones de intervención frente al grupo control.

H2.- Se prevé que buena parte de los participantes presentará ansiedad y depresión, mal afrontamiento de la enfermedad y mal control de los factores de riesgo cardiovascular.

H3.- Se producirán mayores efectos de la intervención sobre las variables en el subgrupo de mujeres, debido a su mayor grado de compromiso con el programa de intervención.

3.3 Objetivos

I. Objetivo general

- Analizar la efectividad de la adición del entrenamiento de la musculatura inspiratoria a un programa de rehabilitación cardiaca de personas con cardiopatía isquémica revascularizadas percutáneamente tras 16 sesiones de intervención, en base a la capacidad funcional, además de la fuerza muscular, soporte social, ansiedad, depresión, afrontamiento de la enfermedad, disfunción sexual, calidad de vida, calidad del sueño, hábitos alimenticios y composición corporal.

II. Objetivos secundarios

- Conocer el perfil biopsicosocial de esta población en base a la capacidad funcional, además de la fuerza muscular, soporte social, ansiedad, depresión, afrontamiento de la enfermedad, disfunción sexual, calidad de vida, calidad del sueño, hábitos alimenticios y composición corporal.

- Analizar las diferencias de género a través de un análisis de subgrupo.

4.- Metodología

Las características del ensayo clínico propuesto son reportadas de acuerdo con las normas SPIRIT (Standard Protocol Items: Recommendations for Interventional Trials)[26].

Diseño del estudio

Ensayo clínico aleatorizado y controlado (en paralelo), triple ciego y de bajo riesgo.

Ámbito de estudio

El estudio se llevará a cabo en la Unidad de Rehabilitación Cardiaca (URC) del Hospital Universitario Virgen de la Victoria de Málaga (HUVV).

Sujetos de Estudio

Personas con CI derivadas a la URC del HUVV a las que se les ha realizado una ACTP.

Criterios de Inclusión: i) personas con CI con ACTP ii) Ambos sexos; iii) Mayores de 18 años; iv) Menores de 80 años; v) Ausencia de déficit cognitivo y/o limitaciones físicas que le impida la realización de ejercicio físico o la realización de los cuestionarios necesarios para la participación en el estudio.

Criterios de exclusión: i) Enfermedad o condición que contraindique la realización de ejercicio como miocardiopatía hipertrófica obstructiva severa, estenosis aórtica severa y/o aneurisma disecante de aorta; ii) Pacientes que rechacen el tratamiento o no firmen el consentimiento informado.

Muestreo, tamaño muestral y aleatorización

Se llevará a cabo un muestreo consecutivo con los pacientes que acudan a la URC del HUVV y cumplan los criterios anteriormente descritos.

El tamaño de muestra se calculó para detectar diferencias entre grupos en nuestra variable principal, la capacidad funcional medida mediante el consumo de oxígeno máximo. De acuerdo con el metaanálisis de Fabero-Garrido et al[9]., las personas con cardiopatía isquémica que han llevado a cabo un programa de entrenamiento de la musculatura inspiratoria tienen de media una diferencia de 2.18 ml de O_2/ kg/min con respecto al grupo control y queremos encontrar una desviación de 1 MET (3.5 ml de O_2 /kg/min). Los cálculos se llevaron a cabo con el software estadístico G*Power 3.1, considerando en todos los casos un nivel de confianza del 95% y una potencia del 80%.

En vista de lo anterior, se estableció un tamaño de muestra de 33 sujetos por grupo (66 en total). Además, para minimizar las repercusiones de los posibles abandonos, la muestra total se incrementó en un 10% hasta 72 pacientes, lo que llevará a la inclusión final de 36 pacientes en cada grupo.

La aleatorización de los participantes se realizará a través de un programa informático que genera una secuencia de números al azar.

Intervención

El grupo control realizará el PRC estándar que consiste 16 sesiones de ejercicio multicomponente (cardiovascular y de fuerza). Además, asistirán a una sesión semanal de educación sanitaria y a otra de terapia psicológica grupal.

El grupo de intervención hará el mismo programa que el grupo control al que se le añadirá el EEMI.

Programa de Rehabilitación Cardiaca.

El PRC habitual será realizado y supervisado por el equipo multidisciplinar habitual del HUVV. Está compuesto por un médico cardiólogo, una médica rehabilitadora, una fisioterapeuta, una enfermera y una psicóloga. De forma previa

a la inclusión en el estudio se realizará a cada participante una ergometría simple para determinar su frecuencia cardiaca máxima (FCM), observar su comportamiento ante esfuerzos máximos y descartar condiciones que contraindiquen el ejercicio. Los sujetos de estudio acudirán 3 días a la semana a la sala de rehabilitación cardiaca del hospital. Dos de ellos los dedicarán a las sesiones de ejercicio físico y el tercero a la educación sanitaria y a la sesión grupal de terapia psicológica.

En la sesión de educación sanitaria se tratará un tema distinto cada semana (conocimientos básicos de su patología, toma adecuada de la medicación, realización segura de ejercicio físico, disfunción sexual, alimentación saludable y control de los factores de riesgo cardiovascular).

Por otra parte, aquellos sujetos que sufran disfunción eréctil se les ofrecerá la posibilidad de ser vistos ser atendidos en la consulta de urología. El médico rehabilitador realizará una valoración nutricional mediante bioimpedanciometría y una valoración morfológica del cuádriceps y otra de la grasa abdominal, ambas mediante ecografía. Los participantes con diabetes mal controlada y/u obesidad mórbida acudirán a la consulta del endocrinólogo del equipo de rehabilitación cardiaca.

Las sesiones de entrenamiento durarán aproximadamente 75 minutos. La estructura será la siguiente:

- Recepción de los pacientes, toma de constantes (tensión arterial, frecuencia cardiaca y glucemia en caso de pacientes diabéticos) y colocación de la telemetría.
- Calentamiento
- Ejercicio de Fuerza: Consistirá en distintos ejercicios con mancuernas y gomas elásticas que implican a varias articulaciones. Se trabajará al 50% de 1 repetición máxima (1RM) medida de forma indirecta con el método de las 20 repeticiones máximas (20RM). Se completarán 3 series de 10 repeticiones de los siguientes ejercicios: flexión del codo, la abducción del hombro y de la extensión de la rodilla. El intervalo de descanso entre series será de 30 segundos.
- Ejercicio Cardiovascular: Se realizará en cinta sin fin o en bicicleta estática. Tendrá una duración de 30 minutos. El primer mes se entrenará al paciente al 70% de la frecuencia cardiaca resultante de aplicar la fórmula de Karvonen y el segundo al 80%. La modalidad podrá ser continua o interválica en función de la tolerancia de la persona.
- Relajación y vuelta a la calma: los pacientes permanecerán sentados en sillas durante 5 minutos durante los que se reproducirá un audio de relajación guiada.
- Comprobación de constantes y despedida.

Cada semana se pesará a los pacientes para monitorizar su evolución y descartar posibles efectos adversos del entrenamiento. El programa se extenderá durante 8 semanas hasta completar 16 sesiones de entrenamiento concurrente. A su finalización se repetirán la ergometría, la analítica de sangre y el resto de las mediciones antropométricas, así como los cuestionarios y tests previos. En el anexo 1 se expone el protocolo de valoración previa al ejercicio físico, contraindicaciones y banderas rojas antes, durante y después del ejercicio físico.

Entrenamiento Específico de la Musculatura Inspiratoria.

Para poder determinar la carga de entrenamiento de cada sujeto se medirá previamente su PIM. Mas adelante se indica el procedimiento de medición. La PIM se reevaluará a mitad del programa (al final de la semana 4) para reajustar la carga de entrenamiento.

El entrenamiento se realizará ajustando la carga del dispositivo al 70% del valor de la PIM realizando 1 sesión de entrenamiento diario 4 días a la semana (lunes, miércoles, viernes y domingo) de 3 series de 10 repeticiones, con un descanso de 3 minutos entre series, durante las 8 semanas que dura el programa de RHB cardiaca. Se utilizará el dispositivo de entrenamiento de musculatura espiratoria EMST 75 LITE con adaptador inspiratorio IA 150 que lo convierte en dispositivo de entrenamiento de la musculatura inspiratoria. Es un producto sanitario clase I del fabricante Aspire Products, LLC. La carga de trabajo es ajustable y se puede variar dentro de un rango que va de 0 a 75 cm de H2O. Se adjunta ficha técnica del producto con fotos en el Anexo 3. Los productos sanitarios clasificados dentro de la clase I se consideran los de menor riesgo y presentan un mínimo daño potencial para pacientes o usuarios.

Uno de los fisioterapeutas del PRC enseñará a los participantes a utilizar el dispositivo de EEMI el primer día que acudan al PRC. Se les indicará que lo realicen en casa. Se les entregará un diario para que registren su cumplimiento. Así mismo se les preguntará semanalmente si tienen alguna duda sobre la utilización del dispositivo. Además, una vez a la semana el fisioterapeuta supervisará de forma presencial la ejecución del EEMI por parte de los participantes.

Variables de estudio, mediciones e instrumentos de medida.

Una vez firmado el consentimiento informado, se recogerán las siguientes variables demográficas: edad, sexo biológico, estado civil, nivel educativo, profesión y nacionalidad.

En la consulta de rehabilitación cardiaca se realizará una anamnesis en la que se recogerán los siguientes datos para la historia clínica y para el estudio: comorbilidades cardíacas (revascularización, IC, cirugía de válvulas cardíacas), presencia de factores de riesgo cardiovascular (tabaquismo actual o previo, hipertensión arterial, dislipidemia, diabetes mellitus, sedentarismo, apnea obstructiva del sueño), comorbilidades del aparato locomotor, neurológicas, vasculares o respiratorias, hábitos de ejercicio físico (tipo y tiempo) y se realiza una estratificación del riesgo cardiaco del paciente (bajo, medio o alto).

Además, antes y después de la Intervención se harán las siguientes valoraciones y evaluaciones.

Se calculará el Índice de comorbilidad de Charlson (Charlson Comorbidity Index en inglés). Se trata de una herramienta validada en español que evalúa la esperanza de vida a diez años. Incluye 19 ítems que, si están presentes, se ha demostrado que afectan de manera específica la esperanza de vida del sujeto. Inicialmente se adaptó para evaluar la supervivencia a un año, pero finalmente se ajustó para evaluar la supervivencia a diez años[27,28].

Para valorar la capacidad funcional, se realizará una ergometría simple siguiendo las recomendaciones de la Sociedad Española de Cardiología[29] en la que se medirá el consumo de Oxígeno máximo (VO_2max), el tiempo de duración de la prueba y el tipo de respuesta. La ergometría, también conocida como prueba de esfuerzo, es además de una técnica diagnóstica que analiza la respuesta del corazón ante el ejercicio, una prueba que es capaz de cuantificar el consumo de oxígeno del individuo que la realiza. En este estudio se realizará en tapiz rodante. Se utilizará el protocolo de Bruce que aumenta progresivamente la velocidad de la cinta y su inclinación cada 3 minutos. Será llevada a cabo por un cardiólogo de la unidad. Se obtendrá el VO_2max en METS (un equivalente metabólico 1 MET = 3,5 ml O_2/kg/min). También se recogerá el tiempo y el tipo de respuesta. Esta podrá ser clínicamente positiva si el cardiólogo aprecia la aparición de angina con el esfuerzo o eléctricamente positiva si aparece una depresión horizontal o descendente del segmento ST $\geq$ 1mm medida a 80ms del punto J. Será negativa en el caso de que no se produzca ninguna de las situaciones anteriores.

Se evaluará el nivel de actividad física mediante el cuestionario RAPA[30](Rapid Assessment of Physical Activity) validado en español[31]. Se diseñó para medir esta variable en adultos mayores. Consta de 9 ítems, siete de ellos buscan determinar si las personas cumplen la recomendación de realizar 30 minutos o más de actividad física moderada, al menos 5 días a la semana. Los dos ítems adicionales miden si las personas realizan ejercicios de flexibilidad y fuerza. En función de la puntuación total establece las siguientes categorías de actividad física: "Sedentario", "Poco activo", "Poco activo regular ligero", "Poco activo regular" y "Activo".

La fracción de eyección del ventrículo izquierdo (FEVI) será medida por el cardiólogo mediante ecografía siguiendo las recomendaciones de la Sociedad Americana de Ecocardiografía parar su uso en ensayos clínicos y también de acuerdo con cardiólogos expertos[32,33].

Para medir la PIM y la presión espiratoria máxima (PEM), se utilizará el manómetro portátil digital MicroRPM® (Vyaire Medical GmbH, Hoechberg, Germany). Las evaluaciones de PIM y Presión Espiratoria Máxima (PEM) se realizarán de acuerdo con las recomendaciones de la Sociedad Torácica Americana (ATS)[34] y de la Sociedad Respiratoria Europea (ERS)[35] y siguiendo el protocolo de la Sociedad Española de Neumología y Cirugía Torácica (SEPAR)[36] La debilidad muscular inspiratoria se considerará con valores inferiores a 62 cmH_2O para las mujeres y de 83 cmH_2O para hombres y la espiratoria por debajo de 81 cmH_2O en mujeres y 109 cmH_2O en hombres[37]. La PIM se reevaluará a mitad del programa (al final de la semana 4) para reajustar la carga de entrenamiento.

Para valorar la calidad de vida, se administrará el cuestionario SF-12 versión 2 (SF-12v2) traducido al español y validado en población de Cataluña[38]. Consta de 12 preguntas, en torno a 8 dimensiones: autopercepción general de salud, capacidad física, funcionamiento físico, rol emocional, funcionamiento social, salud mental, dolor físico y autopercepción de vitalidad con opciones. Mide 2 componentes principales, la salud física (PCS) y la salud mental (MSC). Las puntuaciones de PCS y MCS están normalizadas en una escala de 0 a 100, donde 50 es la media de la población general. puntuaciones más altas indican mejor calidad de vida.

La ansiedad y la depresión se valorarán mediante la escala hospitalaria de ansiedad y depresión (HADS), compuesta por 14 preguntas, se divide en dos subdimensiones: ansiedad (HADS-A) y depresión (HADS-D)[39]. Cada pregunta tiene respuestas de tipo Likert, con puntuaciones que varían de 0 a 3. Por lo tanto, cada subescala tiene una puntuación que va de 0 a 21 puntos. Puntuaciones entre 0 y 7 indican la ausencia de depresión y/o ansiedad, puntuaciones entre 8 y 10 sugieren un trastorno clínicamente significativo, y puntuaciones entre 11 y 21 señalan la presencia de depresión y/o ansiedad de moderada a grave.

Para evaluar la calidad del sueño, se utilizará el índice de calidad del sueño de Pittsburgh[40] (PSQI). Es el cuestionario de autoinforme más utilizado para este fin. Consta de 19 preguntas autoevaluadas cuya valoración global va de 0 a 21. Puntuaciones más elevadas representan una peor calidad del sueño. Por encima de 5 se considera como “mal dormidor” y por debajo como “buen dormidor”. Un cambio de 3 puntos es la diferencia mínima clínicamente significativa.

La escala de Duke[41] en su versión en español[42] se utilizará para evaluar el apoyo social funcional. Se trata de un cuestionario autoadministrado, con una escala de respuesta tipo Likert (1-5). El rango de calificaciones va de 11 a 55 puntos. La puntuación obtenida refleja el apoyo percibido en lugar del apoyo real. La menor puntuación significa menos apoyo. Una puntuación de 32 o más indica un apoyo social generalizado, mientras que una puntuación de menos de 32 indica un apoyo social percibido bajo.

Para valorar la disfunción eréctil en los hombres participantes se utilizará índice el Índice Internacional de Función Eréctil en su versión reducida (IIEF-5)[42] y validada en español[43]. Es un cuestionario de autoevaluación de 5 preguntas que abordan aspectos como la confianza para lograr erecciones, rigidez de las erecciones, capacidad para mantener la erección durante el acto sexual y satisfacción con el acto sexual. Cada pregunta se responde en una escala de 1 a 5, donde 5 es el puntaje más alto y 1 el más bajo. El puntaje total se obtiene sumando los puntajes de cada pregunta, con un rango de 5 a 25. Permite clasificar la disfunción eréctil en leve (17-21), moderada (12-16) o severa (5-11).
Para establecer la severidad de la disnea se utilizará la escala creada por la Asociación del Corazón de Nueva York[44] (en inglés, New York Heart Association, cuyas siglas son NYHA). Refleja la capacidad que tiene el paciente de realizar esfuerzos físicos por eso también se la llama “clase funcional de la NYHA”, que tiene 4 grados indicando mayor disnea a mayor grado.

La bioimpedancia se utilizará para determinar la composición corporal de los participantes. Funciona enviando una corriente eléctrica leve a través del cuerpo y midiendo la resistencia que ejercen los distintos tejidos a su paso que varía según su contenido de agua y grasa. La medición se hará con el bioimpedanciómetro marca Akern modelo Nutrilab. Además, se calculará el índice de masa corporal (IMC) de cada individuo tras pesarlo y tallarlo en la sala de entrenamiento. También se medirá la circunferencia de la cintura a nivel del ombligo.

Se realizará una ecografía nutricional[45]. Se utilizará el ecógrafo marca FujiFilm modelo Sonosite SII. Se medirá la grasa subcutánea en el cuádriceps sin contracción, el grosor del músculo recto femoral (distancia anteroposterior), su diámetro transversal, su área en cm^2 y su circunferencia. Del vasto intermedio se medirá su grosor (distancia anteroposterior). En el abdomen sin contracción se hará la medición de la grasa subcutánea total, la subcutánea superficial y la pre-peritoneal.

Se evaluarán los hábitos alimenticios mediante el cuestionario de adherencia a la dieta mediterránea[46]. Consta de 14 preguntas directas sobre el consumo de alimentos principales de la dieta mediterránea, como son: el aceite de oliva, las frutas, las verduras, las legumbres, el pescado, los frutos secos, el vino y las carnes blancas. Las puntuaciones se agrupan en cuatro categorías: alta adhesión (12-14 puntos), media adhesión (8-11,99 puntos), baja adhesión (0-7,99 puntos).

La fuerza muscular estática máxima del cuádriceps se medirá con el paciente sentado en una camilla y la rodilla flexionada a 90º mediante el dinamómetro de mano marca Hogan Health Industries modelo MicroFet 2.

La fuerza de agarre de mano (FAM) se medirá con un dinamómetro hidráulico homologado Jamar^{Tm}. Se evaluará la fuerza isométrica de los músculos de la mano y el antebrazo con el brazo extendido a lo largo del cuerpo realizando una contracción durante al menos 3 segundos. Se realizarán 3 maniobras separadas al menos 20 segundos entre sí. Se considerará el mayor valor obtenido.

Durante toda la intervención se monitorizarán y recogerán todas las Incidencias que se produzcan ya sean eventos cardiológicos o no. Se analizarán y si fuesen atribuibles a la intervención se tomarían las medidas correspondientes.

El número de sesiones a las que ha acudido cada participante se tendrá en cuenta como una variable más para el análisis estadístico.

Enmascaramiento

Ninguno de los participantes sabrá a qué grupo pertenece ya que a todos se les entregará el mismo dispositivo de EEMI. Para el grupo control se fijará una carga de 5 cm de H_2O. Esta intensidad de trabajo está por debajo de la necesaria para conseguir adaptaciones en los músculos inspiratorios por lo que funcionará a modo de placebo. Los evaluadores tampoco conocerán a qué grupo pertenecen los sujetos de estudio ya que no participarán en el proceso de asignación ni de intervención. El análisis se hará de manera independiente ya que se entregarán los datos codificados.

Análisis estadístico

Se emplearán medidas de tendencia central y dispersión para variables numéricas, así como frecuencias relativas para variables categóricas para evaluar la comparabilidad entre grupos. Se examinarán todas las variables relevantes para este propósito. Se realizará un análisis exploratorio de datos para identificar y abordar valores atípicos que puedan afectar los resultados. Se aplicarán correcciones pertinentes para mitigar su influencia.

Para contrastar las diferencias entre el grupo control y experimental, se utilizará la prueba T de Student para muestras independientes o la prueba no paramétrica de Mann-Whitney según la normalidad de los datos, medida con el test de Kolmogorov-Smirnov. Para las comparaciones pre y post intervención, se empleará t de Student para muestras pareadas en caso de normalidad o la prueba de Wilcoxon, en caso contrario. En caso de variables cualitativas, se utilizarán prueba de chi-cuadrado.

Además de los análisis estadísticos mencionados, se explorarán posibles relaciones complejas y no lineales entre variables mediante modelos de regresión o análisis multivariados, como regresión múltiple o análisis de componentes principales. Los análisis se realizarán con el programa estadístico IBM SPSS v.24.

Riesgos previstos y planes de contingencia

Riesgo previsto	Plan de contingencia
No cumplimiento del EEMI por parte de los participantes	Entrega de registro del cumplimiento en papel en forma de diario
Realización incorrecta de la técnica de EEMI	Sesión semanal de refuerzo en el HUVV
Dudas sobre la realización del EEMI por parte de los participantes	La fisioterapeuta que supervisa el ejercicio físico del PRC estará siempre disponible durante las sesiones para aclarar cualquier duda.

Prospectiva

Sería interesante estudiar en futuros ensayos clínicos el entrenamiento específico de la musculatura espiratoria (EEME) en estos pacientes, tanto de forma aislada, como concurrente con la inspiratoria. Existen estudios que han

demostrado sus beneficios en la estabilidad postural y la velocidad de marcha debido al fortalecimiento de la musculatura central del cuerpo[47,48].

También sería interesante medir los efectos a medio y largo plazo del EEMI ya que hasta ahora los estudios existentes no lo han hecho.

A.5.- Justificación de recursos disponibles

La Universidad de Cádiz, a través de su Biblioteca, proveerá los recursos bibliográficos necesarios para el desarrollo de este proyecto de investigación como son el acceso a las principales editoriales y revistas científicas indexadas. El HUVV cederá las instalaciones y materiales que destina a los PRC para la realización de este estudio.

Las entrevistas y algunas de las mediciones previas y posteriores a la intervención se realizarán en la consulta de rehabilitación cardiaca del HUVV. Tiene unas dimensiones de 7m^2 y cuenta con los siguientes materiales:

- Dinamómetro hidráulico homologado Jamar^{Tm}
- Dinamómetro de mano marca Hogan Health Industries modelo MicroFet 2.
- Cinta métrica.
- Bioimpedanciómetro marca Akern modelo Nutrilab.
- Manómetro portátil digital MicroRPM®.

El entrenamiento físico, las sesiones de educación sanitaria, terapias grupales de psicología y algunas mediciones se llevarán a cabo en la sala de rehabilitación cardiaca del HUVV. Tiene una superficie de 80 m^2 y cuenta con el siguiente equipamiento:

- 2 cintas sin fin.
- 6 bicicletas estáticas.
- Mancuernas.
- Gomas elásticas.
- Sistemas de telemetría para los pacientes.
- Ordenador con el software necesario para monitorizar y sus electrocardiogramas y frecuencias cardiacas (sistema Mortara®)
- Monitores donde observar la electrografía en tiempo real.
- Sillas para la toma de constantes y para las sesiones de educación sanitaria y relajación.
- Altavoz y sistema de reproducción para los audios de relajación.
- Tensiómetros, fonendoscopios y glucómetros para controlar las constantes vitales de los pacientes.
- Carro de parada con desfibrilador y camilla en caso de efectos adversos.
- Pantalla plana de 43” para las sesiones de educación sanitaria.

Las ergometrías las realizará un cardiólogo/a y un enfermero/a especializados del servicio de cardiología del HUVV en su laboratorio de fisiología del ejercicio. Este cuenta con los siguientes elementos:

- Cinta sin fin
- Tensiómetro, glucómetro y fonendoscopio.
- Ordenador con el software adecuado para realizar el protocolo de ejercicio incremental.
- Carro de parada con desfibrilador.
- Camilla

El presente proyecto está respaldado teórica y técnicamente por el grupo de Investigación TIC-256 Intelligent Social Knowledge Based Systems (IntellSOK) y el Observatorio del Dolor de la Universidad de Cádiz. Ambas entidades cuentan con dilatada experiencia en el desarrollo de estudios de investigación y cuentan con los recursos técnicos y conocimientos necesarios para la correcta consecución del proyecto propuesto.

A.6.- Planificación temporal de trabajo

Se estima una duración aproximada total del ensayo clínico de 6 meses para reclutar y finalizar la intervención de los 72 participantes.

Figura 1. Esquema de reclutamiento, intervenciones, y evaluaciones.

	PERIODO DE ESTUDIO			
	Reclutamiento	Asignación	Etapa Posterior a la asignación	
**MOMENTO**	t_{-1}	0	t_0	t_x
**RECLUTAMIENTO:**				
Cribado de selección	X			
Consentimiento informado	X			
Asignación		X		
**INTERVENCIONES:**				
Grupo Experimental PRC estándar			◆———	———◆
Grupo Control PRC+EEMI			◆———	———◆
**EVALUACIONES:**				
Variables sociodemográficas: edad, sexo biológico, estado civil, nivel educativo, nivel de ingresos, profesión, nacionalidad.			X	X
Variables anamnesis: comorbilidades, factores de riesgo, estratificación, Índice de Charlson.			X	X
Variables pre y post intervención:				
METS en ergometría (VO_2max)			X	X
Tiempo de ejercicio ergometría			X	X
Tipo de respuesta en la ergometría			X	X
FEVI			X	X
PIM			X	X
PEM			X	X

Fuerza estática del cuádriceps			X	X
Fuerza de agarre de la mano			X	X
Ecografía nutricional			X	X
Bioimpedancia			X	X
Índice de masa corporal			X	X
Circunferencia de cintura			X	X
Calidad de vida (SF-12)			X	X
Calidad de vida (EuroQol-5D)			X	X
Calidad de vida (Heart Qol)			X	X
Ansiedad y depresión HADS			X	X
Actividad física (RAPA)			X	X
Disnea (NYHA)			X	X
Calidad del sueño (Pittsburg)			X	X
Apoyo social funcional (Duke)			X	X
Disfunción sexual (IIEF-5)			X	X
Cuestionario femenino de disfunción sexual			X	X
Incidencias cardiológicas o no			◆————	————◆
Grado de Cumplimiento				X

***t_{-1}**, realizado en entorno clínico; **0**, realizado previo a la 1ª sesión; **t_0**, evaluación inicial y toma de contacto presencial; **t_1**, 8 semanas; **t_2**, 16 semanas, **t_3**, 24 semanas; **t_x** post-intervención.*

Abreviaturas**: **PRC** programa de rehabilitación cardiaca, **EEMI** entrenamiento específico de la musculatura inspiratoria, **FEVI** fracción de eyección del ventrículo izquierdo, **PIM** presión inspiratoria máxima, **PEM** presión espiratoria máxima, **RM** repetición máxima, **IMC índice de masa corporal, **HADS** cuestionario de ansiedad y depresión, **RAPA** cuestionario rápido de actividad física, **NYHA** New York heart association**, IIEF-5** índice internacional de función eréctil versión reducida.

A.7.- Implicaciones éticas y/o de bioseguridad de la investigación propuesta.

El presente proyecto seguirá los principios éticos recogidos para las investigaciones médicas en seres humanos según la declaración de Helsinki.

Ya que se van a utilizar datos de sujetos reales es imprescindible garantizar la confidencialidad, privacidad y protección de datos de estos. Para ello, la participación de los sujetos será voluntaria, siendo requisito indispensable firmar el consentimiento informado de participación en el estudio. Los participantes podrán abandonarlo siempre que lo deseasen. Para salvaguardar la identidad de los sujetos se codificarán sus nombres y apellidos en la base de datos.

Se trata de un ensayo clínico de bajo nivel de intervención, ya que el EEMI es una intervención muy segura ya que se realiza de forma habitual en otras poblaciones de pacientes cardiacos y respiratorios crónicos sin haberse reportado efectos adversos relevantes[23,49].

Además, los pacientes serán escrupulosamente estratificados según su riesgo cardiovascular y el equipo de rehabilitación cardiaca del HUVV cuenta con una amplia experiencia en el entrenamiento de este tipo de pacientes y sus profesionales están en constante formación.

Por todo lo anterior, el presente ensayo clínico cumple con el epígrafe 3 del artículo 2.2.3 del Reglamento Europeo 536/2014, y 2.4 del Proyecto de Real Decreto por el que se regulan los ensayos clínicos con medicamentos, los Comités de Ética de la Investigación con medicamentos y el Registro de Estudios Clínicos (https://eur-lex.europa.eu/legal-content/ES/TXT/HTML/?uri=CELEX:32014R0536&from=ES): "los procedimientos complementarios de diagnóstico o seguimiento entrañan un riesgo y carga adicionales para la seguridad de los sujetos que son mínimos comparados con los de la práctica clínica habitual."

Los daños y perjuicios irrogados a los participantes se verán satisfechos por el aseguramiento propio, individual o colectivo, que el investigador en cuestión tuviese ya contratado para el ejercicio habitual de su profesión. Así lo recoge el artículo 9.4 del Proyecto de Real Decreto, en desarrollo de lo plasmado en el artículo 76.3 del Reglamento.

Los datos personales únicamente serán utilizados con el propósito del desarrollo del proyecto y sólo serán accesibles para el personal autorizado. La totalidad de la información será utilizada de acuerdo con la Directiva Europea 95/46/CE, según el Reglamento (UE) 2016/679 del Parlamento Europeo y del Consejo de 27 de abril de 2016 en referencia a la protección y a la libre circulación de datos personales de las personas físicas, la Ley Orgánica 15/1999 (13 de diciembre) sobre la Protección de Datos, el Real Decreto 1720/2007 (21 de diciembre) por el que se aprueba el reglamento de desarrollo de la Ley Orgánica 15/1999, y la Ley Orgánica 3/2018, de 5 de diciembre, de Protección de Datos Personales y garantía de los derechos digitales.

Para publicar los resultados del proyecto en revistas internacionales revisadas por pares y presentar los resultados en congresos de todo el mundo, se seguirá la declaración Consolidated Standards of Reporting Trials (CONSORT)50. Además, como parte de la estrategia de traducción de conocimientos, se difundirán los resultados en sitios web institucionales y medios sociales, contactaremos con organizaciones de noticias e identificaremos instituciones asociadas interesadas en los resultados.

A.8.- Bibliografía.

1. Cardiopatía Isquémica - Fundación Española del Corazón [Internet]. [citado 11 de abril de 2024]. Disponible en: https://fundaciondelcorazon.com/informacion-para-pacientes/enfermedades-cardiovasculares/cardiopatia-isquemica.html
2. Bentzon JF, Otsuka F, Virmani R, Falk E. Mechanisms of plaque formation and rupture. Circ Res. 19 de junio de 2014;114(12):1852-66.
3. Alders DJC, Johan Groeneveld AB, Binsl TW, van Beek JHGM. Progressively heterogeneous mismatch of regional oxygen delivery to consumption during graded coronary stenosis in pig left ventricle. Am J Physiol Heart Circ Physiol [Internet]. 2015 [citado 3 de julio de 2024];309(10):H1708-19. Disponible en: www.ajpheart.org
4. Lee JA, Allen DG. Mechanisms of acute ischemic contractile failure of the heart: Role of intracellular calcium. Journal of Clinical Investigation. 1991;88(2):361-7.
5. Todorova M. STUDY OF THE QUALITY OF LIFE WITH REGARD TO THE HEALTH OF PATIENTS WITH CHRONIC ISCHEMIC HEART DISEASE. Vol. 28, KNOWLEDGE-International Journal. 2018.
6. Lietava J, Vohnout B, Penz P, Kuka P, Bucová M, Kosmálová V, et al. Relationship of self-reported exercise tolerance with inflammatory markers in women with stable ischemic heart disease. Neuroendocrinology Letters [Internet]. 2012 [citado 3 de julio de 2024];33(2):50-4. Disponible en: www.nel.edu
7. Vedin O, Lam CSP, Koh AS, Benson L, Teng THK, Tay WT, et al. Significance of Ischemic Heart Disease in Patients with Heart Failure and Preserved, Midrange, and Reduced Ejection Fraction: A Nationwide Cohort Study.

Circ Heart Fail [Internet]. 1 de junio de 2017 [citado 3 de julio de 2024];10(6). Disponible en: http://circheartfailure.ahajournals.org/lookup/suppl/doi:10.1161/CIRCHEARTFAILURE.117.003875/-/DC1.

8. Westin L, Carlsson R, Israelsson B, Willenheimer R, Cline C, Mc Neil TF. Quality of life in patients with ischaemic heart disease: a prospective controlled study. J Intern Med [Internet]. 1997 [citado 3 de julio de 2024];242:239-47. Disponible en: https://onlinelibrary.wiley.com/doi/10.1046/j.1365-2796.1997.00203.x

9. Fabero-Garrido R, Del Corral T, Plaza-Manzano G, Sanz-Ayan P, Izquierdo-García J, López-De-Uralde-Villanueva I. Effects of Respiratory Muscle Training on Exercise Capacity, Quality of Life, and Respiratory and Pulmonary Function in People With Ischemic Heart Disease: Systematic Review and Meta-Analysis. PTJ: Physical Therapy & Rehabilitation Journal | Physical Therapy [Internet]. 2024; Disponible en: https://doi.org/10.1093/ptj/pzad164

10. Elosua R, Sayols-Baixeras S. Genética de la cardiopatía isquémica: del conocimiento actual a las implicaciones clínicas. Rev Esp Cardiol [Internet]. 1 de septiembre de 2017 [citado 27 de junio de 2024];70(9):754-62. Disponible en: http://www.revespcardiol.org/es-genetica-cardiopatia-isquemica-del-conocimiento-articulo-S0300893217302087

11. Wang W, Hu M, Liu H, Yang H, Cai J, Correspondence HL. Global Burden of Disease Study 2019 suggests that metabolic risk factors are the leading drivers of the burden of ischemic heart disease. Cell Metab [Internet]. 2021 [citado 27 de junio de 2024];33:1943-1956.e2. Disponible en: https://doi.org/10.1016/j.cmet.2021.08.005

12. Mehta PK, Wei J, Wenger NK. Ischemic heart disease in women: A focus on risk factors. Vol. 25, Trends in Cardiovascular Medicine. Elsevier Inc.; 2015. p. 140-51.

13. Viéitez Flórez JM, Rivas SG, Zamorano Gómez JL. Cardiopatía isquémica: concepto, clasificación, epidemiología, medidas preventivas y tratamiento no farmacológico.

14. Khan MA, Hashim MJ, Mustafa H, Baniyas MY, Suwaidi SKBM Al, AlKatheeri R, et al. Global Epidemiology of Ischemic Heart Disease: Results from the Global Burden of Disease Study. Cureus [Internet]. 23 de julio de 2020 [citado 1 de julio de 2024];12(7). Disponible en: https://www.cureus.com/articles/36728-global-epidemiology-of-ischemic-heart-disease-results-from-the-global-burden-of-disease-study

15. Vaduganathan M, Mensah GA, Turco JV, Fuster V, Roth GA. The Global Burden of Cardiovascular Diseases and Risk: A Compass for Future Health. Vol. 80, Journal of the American College of Cardiology. Elsevier Inc.; 2022. p. 2361-71.

16. Heart Association A. 2022 Heart Disease & Stroke Statistical Update Fact Sheet Global Burden of Disease. [citado 11 de abril de 2024]; Disponible en: https://www.ahajournals.org/doi/10.1161/CIR.0000000000001052

17. Ambrosetti M, Abreu A, Corrà U, Davos CH, Hansen D, Frederix I, et al. Secondary prevention through comprehensive cardiovascular rehabilitation: From knowledge to implementation. 2020 update. A position paper from the Secondary Prevention and Rehabilitation Section of the European Association of Preventive Cardiology. Jessa Hospital Campus Virga Jesse [Internet]. [citado 3 de julio de 2024];14. Disponible en: https://academic.oup.com/eurjpc/article/28/5/460/6145619

18. Moghei M, Turk-Adawi K, Isaranuwatchai W, Sarrafzadegan N, Oh P, Chessex C, et al. Cardiac rehabilitation costs. Int J Cardiol [Internet]. 1 de octubre de 2017 [citado 3 de julio de 2024];244:322-8. Disponible en: http://www.internationaljournalofcardiology.com/article/S0167527317332291/fulltext

19. Shields GE, Wells A, Doherty P, Heagerty A, Buck D, Davies LM. Cost-effectiveness of cardiac rehabilitation: a systematic review. Heart [Internet]. 2018 [citado 3 de julio de 2024];104:1403-10. Disponible en: http://heart.bmj.com/

20. Kabboul NN, Tomlinson G, Francis TA, Grace SL, Chaves G, Rac V, et al. Comparative effectiveness of the core components of cardiac rehabilitation on mortality and morbidity: A systematic review and network meta-analysis. Vol. 7, Journal of Clinical Medicine. MDPI; 2018.

21. Hossein Pour AH, Gholami M, Saki M, Birjandi M. The effect of inspiratory muscle training on fatigue and dyspnea in patients with heart failure: A randomized, controlled trial. Jpn J Nurs Sci [Internet]. 1 de abril de 2020 [citado 9 de mayo de 2024];17(2). Disponible en: https://pubmed.ncbi.nlm.nih.gov/31429207/

22. Aliu M, Xhema J, Miftari S. Effectiveness of Inspiratory Muscle Training among Chronic Obstructive Pulmonary Disease Patients: A Systematic Review.

23. Ammous O, Feki W, Lotfi T, Khamis AM, Gosselink R, Rebai A, et al. Inspiratory muscle training, with or without concomitant pulmonary rehabilitation, for chronic obstructive pulmonary disease (COPD). Vol. 2023, Cochrane Database of Systematic Reviews. John Wiley and Sons Ltd; 2023.

24. Azambuja ADCM, De Oliveira LZ, Sbruzzi G. Inspiratory Muscle Training in Patients With Heart Failure: What Is New? Systematic Review and Meta-Analysis. Phys Ther [Internet]. 1 de diciembre de 2020 [citado 9 de mayo de 2024];100(12):2099-109. Disponible en: https://pubmed.ncbi.nlm.nih.gov/32936904/

25. Taylor RS, Brown A, Ebrahim S, Jolliffe J, Noorani H, Rees K, et al. Exercise-based rehabilitation for patients with coronary heart disease: Systematic review and meta-analysis of randomized controlled trials. American Journal of Medicine. 15 de mayo de 2004;116(10):682-92.

26. Chan AW, Tetzlaff JM, Altman DG, Laupacis A, Gøtzsche PC, Krleža-Jerić K, et al. Declaración SPIRIT 2013: definición de los elementos estándares del protocolo de un ensayo clínico * [SPIRIT 2013 Statement: defining standard protocol items for clinical trials] [Internet]. Disponible en: www.acponline.org/authors/icmje/ConflictOfInterestForms.do?

27. Charlson ME, Pompei P, Ales KL, Mackenzie CR. A NEW METHOD OF CLASSIFYING PROGNOSTIC COMORBIDITY IN LONGITUDINAL STUDIES: DEVELOPMENT AND VALIDATION. J zyxwvutsrqponmlkjihgfedcbaZYXWVUTSRQPONMLKJIHGFEDCBA Chron Dis. 1987;40(5):373-83.

28. Charlson ME, Charlson RE, Peterson JC, Marinopoulos SS, Briggs WM, Hollenberg JP. The Charlson comorbidity index is adapted to predict costs of chronic disease in primary care patients. J Clin Epidemiol. 1 de diciembre de 2008;61(12):1234-40.

29. Rev Esp Cardiol. Guías de práctica clínica de la Sociedad Española de Cardiología en pruebas de esfuerzo [Internet]. Revista Española de Cardiología. 2000 [citado 21 de mayo de 2024]. p. 1063-94. Disponible en: https://www.revespcardiol.org/es-pdf-X0300893200108041

30. Topolski TD, LoGerfo J, Patrick DL, Williams B, Walwick J, Marsha Patrick MB, et al. The Rapid Assessment of Physical Activity (RAPA) Among Older Adults. [citado 28 de mayo de 2024]; Disponible en: www.cdc.gov/pcd/issues/2006/oct/06_0001.htm

31. Guirao i Goris JA. Elaboración y validación de la versión en español europeo de la escala de valoración rápida de actividad física (RAPA). 2012 [citado 28 de mayo de 2024]; Disponible en: https://dialnet.unirioja.es/servlet/tesis?codigo=64908&info=resumen&idioma=SPA

32. Gottdiener JS, Bednarz J, Devereux R, Gardin J, Klein A, Manning WJ, et al. American Society of Echocardiography recommendations for use of echocardiography in clinical trials: A report from the american society of echocardiography's guidelines and standards committee and the task force on echocardiography in clinical trials. Journal of the American Society of Echocardiography. 2004;17(10):1086-119.

33. Alberto San Román J, Candell-Riera J, Arnold R, Sánchez PL, Aguadé-Bruix S, Bermejo J, et al. Análisis cuantitativo de la función ventricular izquierda como herramienta para la investigación clínica. Fundamentos y metodología [Internet]. Vol. 62, Rev Esp Cardiol. 2009. Disponible en: www.revespcardiol.org

34. ATS/ERS Statement on respiratory muscle testing. Am J Respir Crit Care Med [Internet]. 2002 [citado 29 de marzo de 2024];166(4):518-624. Disponible en: https://pubmed-ncbi-nlm-nih-gov.bibliouca.idm.oclc.org/12186831/

35. Laveneziana P, Albuquerque A, Aliverti A, Babb T, Barreiro E, Dres M, et al. ERS statement on respiratory muscle testing at rest and during exercise. Eur Respir J [Internet]. 1 de junio de 2019 [citado 29 de marzo de 2024];53(6). Disponible en: https://pubmed-ncbi-nlm-nih-gov.bibliouca.idm.oclc.org/30956204/

36. Manual de Procedimientos SEPAR, 4 by SEPAR - Issuu [Internet]. [citado 29 de marzo de 2024]. Disponible en: https://issuu.com/separ/docs/procedimientos4/135

37. Lista-Paz A, Langer D, Barral-Fernández M, Quintela-del-Río A, Gimeno-Santos E, Arbillaga-Etxarri A, et al. Maximal Respiratory Pressure Reference Equations in Healthy Adults and Cut-off Points for Defining Respiratory Muscle Weakness. Arch Bronconeumol [Internet]. 2023 [citado 27 de mayo de 2024];59:813-20. Disponible en: https://doi.org/10.1016/j.arbres.2023.08.016

38. Schmidt S, Vilagut G, Garin O, Cunillera O, Tresserras R, Brugulat P, et al. Normas de referencia para el Cuestionario de Salud SF-12 versión 2 basadas en población general de Cataluña. Med Clin (Barc). 8 de diciembre de 2012;139(14):613-25.

39. Bjelland I, Dahl AA, Haug T, Neckelmann D. The validity of the Hospital Anxiety and Depression Scale An updated literature review.

40. Buysse Charles F Reynolds III DJ, Monk TH, Berman SR, Kupfer DJ. The Pittsburgh Sleep Quality Index: A New Instrument for Psychiatric Practice and Research. Psychiatry Res. 28:193-5.

41. Broadhead WE, Gehlbach SH, De Gruy F V, Kaplan BH. The Duke-UNC Functional Social Support Questionnaire: Measurement of Social Support in Family Medicine Patients. 1988;26(7):709-23.

42. Revilla Ahumada L de la, Bailon Muñoz E, de Dios Luna J, Delgado A, Prados Quel MÁ, Fleitas L. Validación de una escala de apoyo social funcional para su uso en la consulta del médico de familia. Atención primaria: Publicación oficial de la Sociedad Española de Familia y Comunitaria, ISSN 0212-6567, Vol 8, No 9, 1991, págs 688-692 [Internet]. 1991 [citado 28 de mayo de 2024];8(9):688-92. Disponible en: https://dialnet.unirioja.es/servlet/articulo?codigo=7531006

43. Hernández Tatiana Thieme R, Araos F, Hernández RS. Adaptación y Análisis Psicométrico de la Versión Española del Índice Internacional de Función Eréctil (IIEF) en Población Adaptation and Psychometric Analysis of the Spanish Version of the International Index of Erectile Function in Chilean Population. 2017;35:223-30.

44. McMurray JJV, Adamopoulos S, Anker SD, Auricchio A, Böhm M, Dickstein K, et al. ESC Guidelines for the diagnosis and treatment of acute and chronic heart failure 2012: The Task Force for the Diagnosis and Treatment of Acute and Chronic Heart Failure 2012 of the European Society of Cardiology. Developed in collaboration with the Heart Failure Association (HFA) of the ESC. Eur Heart J. 2012;33(14):1787-847.
45. García-Almeida JM, García-García C, Vegas-Aguilar IM, Ballesteros Pomar MD, Cornejo-Pareja IM, Fernández Medina B, et al. Nutritional ultrasound®: Conceptualisation, technical considerations and standardisation. Vol. 70, Endocrinologia, Diabetes y Nutricion. Sociedad Espanola de Endocrinologia y Nutricion; 2023. p. 74-84.
46. Angel Martínez-Gonzá Lez M, García-Arellano A, Toledo E, Salas-Salvadó J, Buil-Cosiales P, Corella D, et al. A 14-Item Mediterranean Diet Assessment Tool and Obesity Indexes among High-Risk Subjects: The PREDIMED Trial. [citado 28 de mayo de 2024]; Disponible en: www.predimed.es,
47. Yamamoto S, Matsunaga A, Wang G, Hoshi K, Kamiya K, Noda C, et al. Effect of Balance Training on Walking Speed and Cardiac Events in Elderly Patients With Ischemic Heart Disease.
48. Lee K, Park D, Lee G. Progressive Respiratory Muscle Training for Improving Trunk Stability in Chronic Stroke Survivors: A Pilot Randomized Controlled Trial. 2019 [citado 15 de mayo de 2024]; Disponible en: https://doi.org/10.1016/j.jstrokecerebrovasdis.2019.01.008
49. Bjarnason-Wehrens B, Predel HG. Inspiratory muscle training-an inspiration for more effective cardiac rehabilitation in heart failure patients? [citado 11 de mayo de 2024]; Disponible en: https://academic.oup.com/eurjpc/article/25/16/1687/5926444
50. Schulz KF, Altman DG, Moher D. CONSORT 2010 Statement: Updated guidelines for reporting parallel group randomised trials. BMC Med [Internet]. 24 de marzo de 2010 [citado 12 de mayo de 2024];8(1):1-9. Disponible en: https://bmcmedicine.biomedcentral.com/articles/10.1186/1741-7015-8-18

ANEXO 1. PROTOCOLO DE VALORACIÓN PREVIA AL EJERCICIO FÍSICO, CONTRAINDICACIONES Y BANDERAS ROJAS ANTES, DURANTE Y DESPUÉS DEL EJERCICIO FÍSICO.

<u>REHABILITACIÓN CARDIACA.PROGRAMA DE ENTRENAMIENTO FÍSICO</u>

TODO PACIENTE ANTES DE INICIAR EJERCICIO FÍSICO, DEBE VALORARSE LAS SIGUIENTES CONSTANTES:

1. **AUSENCIA DE SINTOMATOLOGÍA ASOCIADA**

2. **TOMA DE TEMPERATURA: si Tª igual o >37º C: NO REALIZA EJERCICIO FÍSICO**

3. **CONTROL T.A. BASAL:**
    - La TA basal debe estar <140/90 mmHg.
    - Ante TA >140/90mmHg:
        - Repetir toma TA
        - Si persiste HTA: mantener en reposo 5-10 minutos y volver a tomar TA
        - Si normal: realiza ejercicio físico
        - Si alterada: no realiza ejercicio físico y consultar con el médico

4. **GLUCEMIA:**
    - Ante paciente **<u>diabético en tto con insulina</u>**, se debe
        - MEDIR LA GLUCOSA ANTES Y DESPUÉS DEL EJERCICIO FÍSICO

            - Para evitar el riesgo de hipoglucemia tardía, TOMAR CARBOHIDRATOS DE LENTA ABSORCIÓN (CEREALES., FRUTOS SECOS.) INMEDIATAMENTE DESPUÉS DEL EJERCICIO (en paciente insulinodependiente)

        - **SI GLUCOSA < 80mg,** NO HACER EJERCICIO FÍSICO:
            - Dar carbohidratos de rápida absorción (tableta de glucosa o zumos)

        - **SI GLUCOSA 80mg-100mg:**
            - Dar carbohidratos de rápida absorción (tableta de glucosa o zumos) 15 minutos antes, volver a tomar la glucemia y si valor >150 mg, realizar entrenamiento físico, controlando glucemias en cada componente del entrenamiento.

            - Modificar tratamiento con insulina:

Reducir 2 UI de insulina basal cada 2-3 días si glucemia en ayunas es <130mg, hasta un total de 10 UI.

        - **SI GLUCOSA 250-300mg/dl:**
            - Si el paciente **se encuentra asintomático**: VALORAR CUERPOS CETÓNICOS:
                - Si cuerpos cetónicos >0,6 mmol/l: no ejercicio físico
                - Si cuerpos cetónicos < 0,6 mmol/l: sí ejercicio

        - **SI GLUCOSA > 300mg/dl: NO HACER EJERCICIO FÍSICO** (CONSULTAR CON EL MÉDICO PARA CAMBIOS EN TTO)

CONTRAINDICACIONES PARA EL EJERCICIO FÍSICO

CONTRAINDICACIONES NO ESPECÍFICAS DE LA DIABETES:

- Evidencia de enfermedad cardiovascular no controlada.
- Enfermedad tromboembólica reciente.
- HTA no controlada (TAS > 180 mmHg o TAD > 105 mmHg)
- FC en reposo > 120 latidos/minuto.
- Enfermedad metabólica no controlada (hipo-hipertiroidismo, etc.).
- Alteraciones electrolíticas (hipokalemia, hipomagnesemia, etc.).
- Infecciones agudas o crónicas (hepatitis, SIDA).
- Enfermedades neuromusculares, musculoesqueléticas o reumáticas.

- Gestación complicada.

CONTRAINDICACIONES ESPECÍFICAS DE LA DIABETES:

- Glicemia > de 300 mg/di y/o presencia de cetonuria.
- Hipoglicemia hasta su total recuperación.
- Retinopatía proliferativa no tratada.
- Hemorragia vítrea o hemorragia retiniana reciente importante.
- Neuropatía autonómica severa.
- Neuropatía periférica severa.
- Nefropatía diabética.

BANDERAS ROJAS EN INSUFICIENCIA CARDIACA

NO HACER EJERCICIO ANTE LO SIGUIENTE Y CONSULTAR CON EL MÉDICO;

1. Aumento de peso de 1,3 kg desde la última sesión o 2,2 kg en una semana
2. Empeoramiento de la disnea (o presencia de disnea de reposo)
3. Fatiga excesiva
4. Hinchazón de piernas o abdomen
5. Tos productiva
6. Aumento miccional, sobre todo nicturia
7. Dificultad para dormir por problemas respiratorios (ortopnea, DPN)
8. Dificultad para concentración
9. Descarga de un DAI

BANDERAS ROJAS GENERALES:

NO HACER EJERCICIO ANTE LA SIGUIENTE SINTOMATOLOGÍA Y CONSULTAR CON EL MÉDICO:

1. Dolor en el pecho
2. Mareos
3. Arritmia no conocida ni controlada
4. Hipoglucemia

ANEXO 2. PROTOCOLO VALORACIÓN REHABILITACIÓN CARDIACA

<table>
<tr><th colspan="2">PRE- PROGRAMA</th></tr>
<tr><th>ENFERMERÍA</th><th>FISIOTERAPIA</th></tr>
<tr><td rowspan="3">- Peso, altura, IMC, perímetro abdominal
- Bioimpedanciometría
- Entregar cuestionarios:
- Cuestionario de calidad de vida: SF-12
- HAD
- RAPA
- Disfunción sexual
- Adherencia dieta mediterránea (nutricionista).
- Hoja de medicación.
- Sacar analítica (6 meses).
- ECG basal</td><td>- 6MWT (cinta).
- Dinamometría de mano (Jamar),
- 20 RM deltoides, bíceps, cuádriceps.
- PIM/PEM</td></tr>
<tr><th>MEDICINA</th></tr>
<tr><td>- Ecografía nutricional
- Dinamometría cuádriceps, bíceps y deltoides
- Bioimpedanciometría
- PIM / PEM</td></tr>
</table>

<table>
<tr><th colspan="2">POST- PROGRAMA</th></tr>
<tr><th>ENFERMERÍA</th><th>FISIOTERAPIA</th></tr>
<tr><td rowspan="3">- IMC, perímetro abdominal
- Entregar cuestionarios:
- Cuestionario de calidad de vida: SF-12
- HAD
- RAPA
- Disfunción sexual
- Adherencia dieta mediterránea (nutricionista).
- Redactar Informe final de enfermería
- Entregar sobres</td><td>- 6MWT (cinta).
- Dinamometría: mano (Jamar).
- PIM/PEM</td></tr>
<tr><th>MEDICINA</th></tr>
<tr><td>- Ecografía de nutricional
- Dinamometría cuádriceps, bíceps y deltoides
- Bioimpedanciometría
- PIM / PEM</td></tr>
</table>

Abreviaturas:
IMC: índice de masa corporal
6MWT: test de los 6 minutos marcha
SF-12v2: cuestionario de calidad de vida versión 2
IC: Insuficiencia cardiaca
HAD: escala de depresión y ansiedad hospitalaria
RAPA: cuestionario rápido para la valoración de la actividad física de adultos mayores
ECG: electrocardiograma
20 RM: 20 repeticiones máximas
PIM: presión inspiratoria máxima medida en boca
PEM: presión espiratoria máxima medida en boca

ANEXO 3. DISPOSITIVOS PARA ENTRENAR LA MUSCULATURA INSPIRATORIA (los dos dispositivos, cuyas fichas técnicas se aportan a continuación, se acoplan para entre sí para formar la válvula de entrenamiento de la musculatura inspiratoria)

EMST75 LITE. Entrenador Fuerza Muscular Espiratoria

PRESENTACIÓN:

**EMST75 LITE**	**Ref.**	**798190166469**
	**EAN**	**0798190166469**

El EMST75 LITE es un dispositivo médico de presión espiratoria positiva (PEP). Está indicado para personas con debilidad muscular respiratoria progresiva, como ELA, Parkinson y esclerosis múltiple; pacientes con enfermedad pulmonar obstructiva crónica (EPOC); cáncer de cabeza y cuello; ictus; lesiones de la médula espinal; y otras afecciones que provocan un aclaramiento anormal de las vías respiratorias y una producción de tos debilitada.

El dispositivo es pequeño, ligero y fácil de usar, lo que lo hace ideal para uso doméstico en casa o de viaje.

DESCRIPCIÓN

Está formado por un tubo de plexiglás de color verde que contiene un muelle de presión ajustable que controla una válvula flexible. En uno de los extremos tiene una boquilla por la cual el paciente debe espirar y dispone de un orificio cerrado por una válvula flexible que impide el paso de aire a través del mismo.

La presión ejercida por el muelle puede regularse girando el eje central. Sobre el cuerpo exterior hay grabada una escala de medición indicada en cm de H_2O y en escala de colores. Al girar el eje, aumentando o disminuyendo la presión que el muelle ejerce sobre la válvula, puede regularse la fuerza necesaria para que la válvula abra o cierre el paso de aire a su través. Dispone de diferentes niveles de presión que permiten controlar el progreso del paciente.

Se entrena simplemente soplando en el dispositivo para superar el umbral de presión. Cuando genera suficiente presión, la válvula de resorte se abre. Proporciona una carga de presión constante en espiración.

Tiene un diseño ergonómico para un uso cómodo y es compacto y ligero para facilitar su transporte.

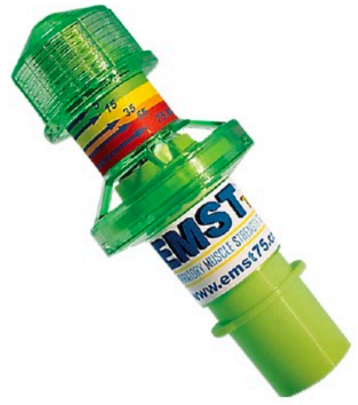

CARACTERÍSTICAS Y MATERIALES

- Marca CE, Clase I, No estéril
- Dispositivo de presión positiva
- Evita la acumulación de secreciones
- Presión regulable de 0 a 75 cm H_2O
- Válvula uní-direccional independiente del flujo de aire
- Fácil de limpiar
- Incorpora pinza nasal
- **Dimensiones**
  - Longitud: 15 cm
  - Diámetro: 6 cm
  - Peso: 70 g
- **Materiales**
  - Cuerpo: Material policarbonato de grado médico y silicona
  - Escala: Impresión en etiqueta
  - Boquilla y Pinza nasal: Polipropileno
- Color: verde

MODO DE EMPLEO

El paciente debe espirar por la boquilla con fuerza suficiente para vencer la resistencia del muelle y conseguir que la válvula abra el paso de aire. Durante el momento de la espiración, cuando no se ha abierto aún la válvula, se genera una presión positiva que ayuda a desprender las secreciones y a despejar las vías respiratorias.

CALIBRACIÓN

El EMST75 Lite es un dispositivo manual calibrado que incluye una válvula unidireccional, accionada por resorte con un dial externo ajustable. La válvula bloquea el flujo de aire hasta que la presión es suficiente. Una vez que se produce la presión deseada, la válvula se abre y el aire comienza a fluir a través del dispositivo. El dial permite ajustar la cantidad de presión en un rango entre 0 y 75 cm H2O. El umbral de presión se basa en la presión espiratoria máxima (PEM) del paciente.

Durante el entrenamiento, el dispositivo de umbral de presión se ajusta de forma incremental para aumentar progresivamente la resistencia (sobrecarga progresiva). La fuerza espiratoria debe ser suficiente para abrir la válvula de resorte y permitir el flujo de aire. La válvula requiere un flujo constante de aire para permanecer abierta. Si la fuerza espiratoria es inadecuada, la válvula no se abrirá y no fluirá aire a través del dispositivo. La "dosis" de EMST se define normalmente en términos de número de repeticiones por serie, con 5 series cada día, durante 5 días a la semana, con la resistencia del aparato fijada en el 75% de la del PEM del paciente y se aumenta cada semana.

Cómo encontrar en el dispositivo la presión que corresponde al 75% de la PEM:

Gire el dial de ajuste verde de modo que el pequeño tornillo se sitúe sobre o justo encima del número 5. Esto es 5cmH20. Aquí es donde usted comienza.
Una vuelta completa aumentará la presión a 15cmH20. El tornillo se asentará sobre del número 15. Una vuelta completa más le llevará a 25cmH20 (el tornillo estará a medio camino entre 15 y 35). Una vuelta completa más le llevará a 35cmH20.
A medida que aumenta la presión en el EMST75, el muelle se tensa y la presión aumenta más por vuelta.
A partir de 35 cmH20, una vuelta completa aumentará la presión en 20 cmH20 hasta 55 cmH20, y una vuelta completa más le llevará al máximo en 75 cmH20. (Las 2 últimas vueltas completas representan 20 cmH20 cada una).

En la tabla siguiente se resume la información de ajuste de presión:

Presión (desde-hasta)	1 vuelta completa =	¼ de vuelta =
5-35 cmH2O	10 cmH2O	2.5 cmH2O
37-75 cmH2O	20 cmH2O	5 cmH2O

INSTRUCCIONES DE LIMPIEZA

- Después de cada sesión, limpie el EMST75 LITE y la boquilla con agua tibia y jabón suave.
- Aclare haciendo pasar agua limpia a través del mismo.
- Sacuda el exceso de agua y déjelo secar al aire.
- No lo exponga a calor o frío extremos.
- Una vez seco, guárdelo hasta la próxima utilización.

CONTIENE

- Dispositivo EMST75 LITE
- 2 boquillas: 1 redonda de 22 mm de diámetro y 1 ovalada
- Pinza nasal
- Instrucciones de uso

PRECAUCIONES Y ADVERTENCIAS

No deje que los niños jueguen con el dispositivo EMST75 LITE.
Si no está seguro de cómo utilizar este producto, consulte con el farmacéutico o a su médico y lea las instrucciones de uso que acompañan al producto.

Busque orientación de su profesional médico para las siguientes condiciones:
- Antecedentes de neumotórax espontáneo.
- Drenaje de LCR (o necesidad de controlar la presión intracraneal).
- Cirugía facial, oral, de cuello, cráneo o tórax reciente (incluida la cirugía cardíaca) o traumatismo.
- Epistaxis.

IA150. Adaptador Inspiratorio para Entrenador Fuerza Muscular Inspiratoria

PRESENTACIÓN:
IA150 — **Ref. 798190096667**
EAN 0798190096667

El adaptador inspiratorio IA150 es un adaptador diseñado para crear un entrenador inspiratorio. Utilice el IA150 con el EMST150 o EMST75 Lite para crear un dispositivo de entrenamiento de la fuerza muscular respiratoria de doble uso. Con la adición del IA150 puede entrenar los músculos inspiratorios con el EMST75 Lite y con los umbrales de presión más altos que solo se encuentran con el EMST150.
El IA150 utiliza los mismos umbrales de presión, de 0 cm de H_2O a 150 cm de H_2O, proporcionando al paciente el mejor rango de ejercicios para sus necesidades individuales.

Simplemente inserte el dispositivo EMST en el IA150 e inmediatamente creará un dispositivo inspiratorio-espiratorio de doble uso.

El dispositivo es pequeño, ligero y fácil de usar, lo que lo hace ideal para uso doméstico en casa o de viaje.

DESCRIPCIÓN

Está formado por un tubo de plexiglás de color azul que se conecta al EMST y lo convierte en un entrenador de fuerza muscular inspiratoria.

La presión ejercida por el muelle puede regularse girando el eje central del EMST y después insertar el dispositivo en el adaptador inspiratorio.

Se entrena simplemente inspirando en el dispositivo para superar el umbral de presión. Cuando genera suficiente presión, la válvula de resorte se abre. Proporciona una carga de presión constante en espiración.

Tiene un diseño ergonómico para un uso cómodo y es compacto y ligero para facilitar su transporte.

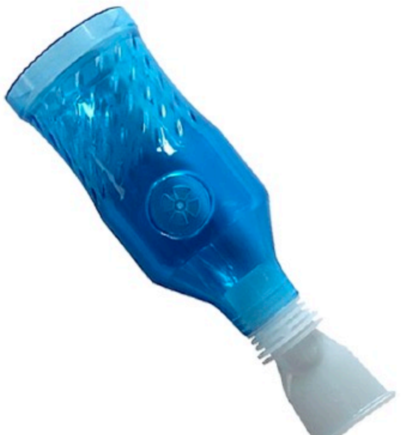

CARACTERÍSTICAS Y MATERIALES

- Marca CE, Clase I, No estéril
- Presión regulable de 0 a 150 cm H_2O
- Válvula uní-direccional independiente del flujo de aire
- Fácil de limpiar
- **Dimensiones**
  - Longitud: 6 cm
  - Diámetro: 6 cm
  - Peso: 22 g
- **Materiales**
  - Cuerpo: Material policarbonato de grado médico y silicona
  - Boquilla: Polipropileno
- Color: azul

MODO DE EMPLEO

El paciente debe inspirar por la boquilla del adaptador con fuerza suficiente para vencer la resistencia del muelle y conseguir que la válvula abra el paso de aire.

INSTRUCCIONES DE LIMPIEZA

- Después de cada sesión, limpie el IA150 y la boquilla con agua tibia y jabón suave.
- Aclare haciendo pasar agua limpia a través del mismo.
- Sacuda el exceso de agua y déjelo secar al aire.
- No lo exponga a calor o frío extremos.
- Una vez seco, guárdelo hasta la próxima utilización.

CONTIENE

- Dispositivo IA150
- Boquilla
- Instrucciones de uso

PRECAUCIONES Y ADVERTENCIAS

No deje que los niños jueguen con el dispositivo IA150.
Si no está seguro de cómo utilizar este producto, consulte con el farmacéutico o a su médico y lea las instrucciones de uso que acompañan al producto.

CONSERVACIÓN

Guarde su IA150 en la caja original o en un envase adecuado mientras no lo utilice.

OBSERVACIONES

Es importante que observe las instrucciones de uso.

FABRICANTE

Aspire Products, LLC
101 VFW ROAD, SUITE 2C
CEDAR POINT, NC 28584
www.emst150.com

DISTRIBUIDOR

Keylab Medical, S.L. C/ Murcia, 27, 08027-Barcelona
Tel. 933 408 553 Fax 933 528 269
www.keylabmedical.com
keylab@keylabmedical.com

Nº REV	MODIFICACIÓN	REDACTADO POR	APROBADO POR	FECHA
01	Redacción Inicial	O.Grau	A.Bamio	09/03/23
02	Modificación	O.Grau	A.Bamio	13/09/23
