# Supplementary material for: Effectiveness of adding inspiratory muscle training to a cardiac rehabilitation program in people with acute myocardial infarction revascularized by percutaneous coronary intervention (CARDIOINSPIRE): Study protocol for a randomized controlled trial
Source: PLoS One. 2026 Mar 10;21(3):e0343947. doi: 10.1371/journal.pone.0343947 (PMC12974859; doi:10.1371/journal.pone.0343947)
Supplement: S3 File — (PDF) [file pone.0343947.s003.pdf]

WRITTEN INFORMED CONSENT

Study title: "Effectiveness of adding inspiratory muscle training to a cardiac rehabilitation program for people with ischemic heart disease revascularized by percutaneous transluminal coronary angioplasty".

Principal investigators:
José María Zuazagoitia de la Lama-Noriega. Department of Nursing and Physiotherapy. University of Cadiz. .josemaria.zuazagoitia@uca.es

Attending physician in charge:
Adela María Gómez González. Virgen de la Victoria University Hospital of Malaga. adelareha@gmail.com

Center: Hospital Universitario Virgen de la Victoria de Málaga.

I (name and surname) ____

- I have read and understood the information sheet given to me.
- I was able to ask questions about the study.
- I have received enough information about the study.
- I have spoken with Dr. Adela María Gómez González Director UGC Medicina Física y Rehabilitación Interniveles del Hospital Universitario Virgen de la Victoria.

I understand that my participation is voluntary. I understand that I can withdraw from the study:

1º Whenever I want
2º Without having to give explanations.
3º Without any repercussions on my medical care.

I freely agree to participate in the study. DATE:

PARTICIPANT'S SIGNATURE

*Two copies of this document must be signed, one for the participant and one for the clinical history.

INFORMATION SHEET FOR THE PARTICIPANT IN A CLINICAL RESEARCH STUDY

Title of the study "Effectiveness of adding inspiratory muscle training to a cardiac rehabilitation program for people with ischemic heart disease revascularized by percutaneous transluminal coronary angioplasty".

Principal investigator:
José María Zuazagoitia de la Lama-Noriega. Department of Nursing and Physiotherapy. University of Cadiz. .josemaria.zuazagoitia@uca.es

Responsible for assistance:
Adela María Gómez González. Virgen de la Victoria University Hospital of Malaga. adelareha@gmail.com

Center: Hospital Universitario Virgen de la Victoria de Málaga.

INTRODUCTION

You are invited to participate in a study that has been approved by the Clinical Research Ethics Committee of the province of Malaga. Please read this information sheet carefully. Dr. Adela María Gómez González, Director of the UGC Physical Medicine and Interlevel Rehabilitation of the Hospital Universitario Virgen de la Victoria, will clarify any doubts you may have.

VOLUNTARY PARTICIPATION

Your participation in this study is voluntary and you may cancel your decision and withdraw your consent at any time without altering your relationship with health care, nor will it affect your treatment or the care you may need.

GENERAL DESCRIPTION OF THE STUDY

Ischemic heart disease or coronary artery disease consists of the narrowing of the internal lumen of the arteries that carry blood to the heart. As the section of the artery decreases, so does the blood flow and therefore the oxygen supply to the heart muscle, which can lead to angina pectoris or even an acute myocardial infarction. It is the leading cause of death from cardiovascular disease, responsible for 9.44 million deaths in 2021 and numerous sequelae in people who survive such an event.
For this reason, it is one of the most important public health problems in all countries of the world, entailing high health and social costs.

The non-pharmacological treatment with the most evidence at present to improve the quality of life of patients and prevent subsequent cardiovascular events is cardiac rehabilitation programs. These include smoking cessation, control of other cardiovascular risk factors, health education, psychological therapy and an exercise program. Two modalities of exercise are performed, cardiovascular or aerobic resistance and strength training. It is not clear today whether adding specific training of the inspiratory musculature to the cardiac rehabilitation programs of people who have the same pathology as you and have undergone the same treatment as you (cardiac catheterization and placement of one or more stents) has benefits. This is why it is not currently used routinely. This is the main reason why this clinical trial is being conducted, to try to increase knowledge on this subject.

This study will analyze: their capacity to exercise, their muscle strength, their social support, whether they have anxiety and/or depression, how they cope with the disease, whether they suffer from any sexual dysfunction, their quality of life, their quality of sleep, their eating habits and their body composition. The aim is also to know the profile of the participants and to find out if there are differences in the response to inspiratory muscle training between men and women. Participants will be given different questionnaires to fill in and will undergo some of the usual tests in cardiac rehabilitation programs.

The specific training of the inspiratory musculature will have to be done by yourself at home once you have been given the appropriate instructions at the hospital. You will be given a device through which you will have to breathe several times during the training. This will consist of doing 3 sets of 10 repetitions 4 days a week, with 3 minutes rest between sets, during the 8 weeks of the cardiac rehabilitation program.

Participants will be divided into two groups, a control group and an intervention group. Neither you nor the people who will supervise you during the program will know which group you belong to. All participants will be given the same device to train the inspiratory muscles. In the control group, the resistance that will be set will be 5% of the maximum inspiratory pressure previously measured for each individual. In the intervention group, resistance will be set at 70% of the maximum inspiratory pressure.

It is expected that some of the aspects to be studied will improve, as this has been the case in similar studies with similar groups of patients.

BENEFITS AND RISKS OF YOUR PARTICIPATION IN THE STUDY

Participation in the study is expected to provide you with the following direct benefits: increased functional capacity, increased inspiratory muscle strength and improved anxiety, depression and quality of life.

Although you may not obtain benefits. We hope that the information we obtain will serve to expand scientific knowledge about inspiratory muscle training in people with ischemic heart disease revascularized by percutaneous transluminal coronary angioplasty and may help others in the future.

FINANCIAL COMPENSATION

Your participation in the study will not involve any expense to you.

CONFIDENTIALITY

Your personal data collected in the performance of this study will be subject to personal data processing, always respecting the provisions of the General Data Protection Regulation of the European Union (GDPR) and the Organic Law 3/2018, of December 5, on the Protection of Personal Data and guarantee of digital rights (LOPDGDD).

The person responsible for the processing of personal data is: Adela María Gómez González. adelareha@gmail.com

The contact details of those responsible are: José María Zuazagoitia de la Lama-Noriegajosemaria.zuazagoitia@uca.es and Adela María Gómez González. adelareha@gmail.com

Legal basis: the processing of personal data is covered by the legal basis established in articles 6.1.a) and 9.2.a) of the RGPD, which establishes that the processing will be lawful if the data subject or his legal representative gave his explicit consent to the processing of his personal data for one or more specific purposes. The data subject or his or her legal representative may give consent to the use of his or her personal data for health research purposes, and in particular biomedical research. The purpose may include categories related to general areas linked to a medical or research specialty, all in accordance with the seventeenth additional provision 2.a) of the LOPDGDD.

Recipients or categories of recipients. Your personal data will be treated with the utmost confidentiality and will not be disclosed to third parties outside the research project. There are no recipients or

categories of recipients of your personal data. In no case will your personal data be subject to international transfer of personal data. If the results of the study are published, your personal data will not be published and your identity will remain anonymous.

Exercising your personal data protection rights. You have the right to request access to your personal data subject to processing from the data controller. You also have the right to rectification of your personal data, to the deletion of your personal data, to the limitation of processing, to oppose the processing, to data portability and not to be subject to decisions based solely on the automated processing of your data, all in accordance with and subject to the limitations provided for in the RGPD and the LOPDGDD for health and biomedical research. These rights will be exercised through the e-mail address: adelareha@gmail.com

You may at any time exercise the right to withdraw your consent to the processing of your personal data, without affecting the lawfulness of the processing for health research purposes based on the consent prior to its withdrawal. This right shall be exercised through the e-mail address: adelareha@gmail.com

You have the right to file a complaint with the supervisory authority, which is the Council for Transparency and Protection of Personal Data of Andalusia, which exercise its competence for the processing of personal data managed by the autonomous institutions of Andalusia, by the autonomous administration, by local administrations, and by other public and private law entities dependent on any of them, as well as by the universities of the Andalusian university system. For all other cases, you have the right to file a complaint with the Spanish Data Protection Agency.

FUNDING

This study does not have specific funding at present, but it is being submitted to regional and national competitive calls for proposals.

WITHDRAWAL OF CONSENT

You may withdraw your consent at any time without explanation. You should also know that you may be excluded from the study if the study investigators deem it appropriate.

Before signing, read the document carefully, ask all the questions you consider appropriate, and if you wish, consult it with as many people as you consider necessary. In case of doubt you should contact Dr. Adela Maía Gómez González.

Signatures:

Signature of the patient:

Name:
Date:

Signature of the Investigator:

Name:
Date:

*Two copies of this document must be signed, one for the participant and one for the medical record.

.
