# Supplementary material for: Effectiveness of adding inspiratory muscle training to a cardiac rehabilitation program in people with acute myocardial infarction revascularized by percutaneous coronary intervention (CARDIOINSPIRE): Study protocol for a randomized controlled trial
Source: PLoS One. 2026 Mar 10;21(3):e0343947. doi: 10.1371/journal.pone.0343947 (PMC12974859; doi:10.1371/journal.pone.0343947)
Supplement: S4 File — (PDF) [file pone.0343947.s004.pdf]

CONSENTIMIENTO INFORMADO POR ESCRITO

Título del estudio: "Efectividad de la adición del entrenamiento de la musculatura inspiratoria a un programa de rehabilitación cardiaca de personas con cardiopatía isquémica revascularizadas mediante angioplastia coronaria transluminal percutánea".

Investigadores principales:
José María Zuazagoitia de la Lama-Noriega. Departamento de Enfermería y Fisioterapia. Universidad de Cádiz. josemaria.zuazagoitia@uca.es.

Responsable asistencial:
Adela María Gómez González. Hospital Universitario Virgen de la Victoria de Málaga. adelareha@gmail.com

Centro: Hospital Universitario Virgen de la Victoria de Málaga

Yo (nombre y apellidos) ____

- He leído y comprendido la hoja de información que se me ha entregado.
- He podido hacer preguntas sobre el estudio.
- He recibido suficiente información sobre el estudio.
- He hablado con la Dra. Adela María Gómez González Directora UGC Medicina Física y Rehabilitación Interniveles del Hospital Universitario Virgen de la Victoria.

Comprendo que mi participación es voluntaria. Comprendo que puedo retirarme del estudio:

1º Cuando quiera
2º Sin tener que dar explicaciones.
3º Sin que esto repercuta en mis cuidados médicos.

Presto libremente mi conformidad para participar en el estudio.

FECHA:

FIRMA DEL PARTICIPANTE

*Se deberán firmar dos copias de este documento, una para el participante y otra para la historia clínica.

HOJA DE INFORMACIÓN AL PARTICIPANTE EN UN ESTUDIO DE INVESTIGACIÓN CLÍNICA

Título del estudio "Efectividad de la adición del entrenamiento de la musculatura inspiratoria a un programa de rehabilitación cardiaca de personas con cardiopatía isquémica revascularizadas mediante angioplastia coronaria transluminal percutánea".

Investigador principal:
José María Zuazagoitia de la Lama-Noriega. Departamento de Enfermería y Fisioterapia. Universidad de Cádiz. josemaria.zuazagoitia@uca.es.

Responsable asistencial:
Adela María Gómez González. Hospital Universitario Virgen de la Victoria de Málaga. adelareha@gmail.com

Centro: Hospital Universitario Virgen de la Victoria de Málaga

INTRODUCCIÓN

Se le invita a participar en un estudio que ha sido aprobado por el Comité Ético de Investigación Clínica de la provincia de Málaga. Por favor, lea esta hoja informativa con atención. La Dra. Adela María Gómez González Directora UGC Medicina Física y Rehabilitación Interniveles del Hospital Universitario Virgen de la Victoria le aclarará las dudas que le puedan surgir.

PARTICIPACIÓN VOLUNTARIA

Su participación en este estudio es voluntaria y puede anular su decisión y retirar el consentimiento en cualquier momento sin que por ello altere su relación con la atención sanitaria, ni se produzca perjuicio en su tratamiento o en la atención que usted pueda necesitar.

DESCRIPCIÓN GENERAL DEL ESTUDIO

La cardiopatía isquémica o enfermedad de las arterias coronarias consiste en el estrechamiento de la luz interna de las arterias que llevan la sangre al corazón. A medida que disminuye la sección de la arteria, también lo hace el flujo de sangre y por tanto el aporte de oxígeno al músculo cardiaco, pudiendo provocar una angina de pecho o incluso un infarto agudo de miocardio. Es la principal causa de muerte por enfermedad cardiovascular, responsable de 9,44 millones de muertes en 2021 y numerosas secuelas en las personas que sobreviven a un evento de este tipo. Por esta razón es uno de los problemas de salud pública más importantes en todos los países del mundo que conlleva un alto gasto sanitario y social.

El tratamiento no farmacológico con más evidencia en la actualidad para mejorar la calidad de vida de los pacientes y además prevenir posteriores eventos cardiovasculares son los programas de rehabilitación cardiaca. Estos incluyen el abandono del tabaco, el control del resto de factores de riesgo cardiovascular, educación sanitaria, terapia psicológica y un programa de ejercicio. Se realizan dos modalidades de ejercicio, el cardiovascular o de resistencia aérobica y el de fuerza. No está claro hoy en día si añadir entrenamiento específico de la musculatura inspiratoria a los programas de rehabilitación cardiaca de personas que tienen su misma patología y les han realizado el mismo tratamiento que a usted (cateterismo cardiaco y colocación de uno o varios stents) tiene beneficios. Por ello no se utiliza actualmente de forma rutinaria. Esta es la razón principal por la que se realiza este ensayo clínico, para tratar de aumentar el conocimiento sobre este tema.

En este estudio se analizará: su capacidad de hacer ejercicio, su fuerza muscular, su soporte social, si tiene ansiedad y/o depresión, cómo afronta la enfermedad, si sufre alguna disfunción sexual, cuál es su calidad de vida, su calidad del sueño, sus hábitos alimenticios y su composición corporal. También se pretende conocer el perfil de los participantes y averiguar si hay diferencias en la respuesta al entrenamiento de la musculatura inspiratoria entre hombres y mujeres. Se le entregarán distintos cuestionarios que tendrá que rellenar y se le harán algunas pruebas habituales en los programas de rehabilitación cardiaca.

El entrenamiento específico de la musculatura inspiratoria lo tendrá que realizar usted por su cuenta en su domicilio una vez que le hayan dado las indicaciones adecuadas en el hospital. Se le entregará un dispositivo por el que tendrá que respirar varias veces cuando haga el entrenamiento. Este consistirá en hacer 3 series de 10 repeticiones 4 días a la semana, con 3 minutos de descanso entre series, durante las 8 semanas que dura el programa de rehabilitación cardiaca.

Se dividirá a los participantes en dos grupos, grupo control y grupo de intervención. Ni usted ni las personas que le supervisarán durante el programa sabrán a cuál de ellos pertenece. A todos los participantes se les entregará el mismo dispositivo para entrenar la musculatura inspiratoria. En el grupo control la resistencia que se fijará será del 5% de la presión inspiratoria máxima que se le haya medido previamente a cada individuo. En el grupo de intervención se fijarán resistencias del 70% de la presión inspiratoria máxima.

Se espera que algunos de los aspectos que se estudiarán mejoren, ya que así ha sucedido en estudios similares con grupos de pacientes parecidos.

BENEFICIOS Y RIESGOS DERIVADOS DE SU PARTICIPACIÓN EN EL ESTUDIO

Se espera que la participación en el estudio le proporcione los siguientes beneficios directos: aumento de su capacidad funcional, incremento de la fuerza de la musculatura inspiratoria y una mejora de su ansiedad, depresión y calidad de vida.

Aunque puede que usted no obtenga beneficios. Esperamos que la información que obtengamos sirva para ampliar el conocimiento científico sobre el entrenamiento de la musculatura inspiratoria en personas con cardiopatía isquémica revascularizadas mediante angioplastia coronaria transluminal percutánea y pueda ayudar a otras personas en el futuro.

COMPENSACIÓN ECONÓMICA

Su participación en el estudio no supondrá ningún gasto para usted.

CONFIDENCIALIDAD

Sus datos personales recogidos en la realización de este estudio serán objeto de un tratamiento de datos personales, respetando siempre lo establecido en el Reglamento General de Protección de Datos de la Unión Europea (RGPD) y la Ley Orgánica 3/2018, de 5 de diciembre, de Protección de Datos de Carácter Personal y garantía de los derechos digitales (LOPDGDD).

El responsable del tratamiento de datos personales es: Adela María Gómez González adelareha@gmail.com

Los datos de contacto de los responsables son: José María Zuazagoitia de la Lama-Noriega josemaria.zuazagoitia@uca.es y Adela María Gómez González adelareha@gmail.com

Base legal: el tratamiento de datos personales se encuentra amparado en la base legal establecida en los arts. 6.1.a) y 9.2.a) del RGPD, que establece que el tratamiento será lícito si el interesado o su representante legal dio su consentimiento explícito para el tratamiento de sus datos personales para uno o varios fines específicos. El interesado o su representante legal puede otorgar su consentimiento para el uso de sus datos personales con fines de investigación en salud, y, en particular, la biomédica. La finalidad puede abarcar categorías relacionadas con áreas generales vinculadas a una especialidad médica o investigadora, todo ello de conformidad con la Disposición adicional decimoséptima 2.a) de la LOPDGDD.

Destinatarios o categorías de destinatarios. Sus datos personales serán tratados con la más absoluta confidencialidad por lo que no serán cedidos a terceras personas ajenas al proyecto de investigación. No existen destinatarios o

categorías de destinatarios de sus datos personales. En ningún caso sus datos personales serán objeto de transferencia internacional de datos personales. Si se publican los resultados del estudio, sus datos personales no serán publicados y su identidad permanecerá anónima.

Ejercicio de los derechos de protección de datos personales. Tiene derecho a solicitar al responsable el acceso a sus datos personales sometidos a tratamiento. Asimismo, tiene derecho a la rectificación de sus datos personales, a la supresión de sus datos personales, a la limitación del tratamiento, a oponerse al tratamiento, a la portabilidad de los datos y a no ser objeto de decisiones basadas únicamente en el tratamiento automatizado de sus datos, todo ello de conformidad y con las limitaciones previstas en el RGPD y en la LOPDGDD para la investigación en salud y biomédica. Estos derechos se ejercerán a través de la dirección de correo electrónico: adelareha@gmail.com

En cualquier momento puede ejercer el derecho a retirar el consentimiento para el tratamiento de sus datos personales, sin que ello afecte a la licitud del tratamiento con fines de investigación sanitaria basado en el consentimiento previo a su retirada. Este derecho se ejercerá a través de la dirección de correo electrónico: adelareha@gmail.com

Tiene el derecho a presentar una reclamación ante la autoridad de control, que es el Consejo de Transparencia y Protección de Datos Personales de Andalucía, que ejercer su competencia para los tratamientos de datos personales gestionados por las instituciones autonómicas de Andalucía, por la Administración autonómica, por las Administraciones locales, y por otras entidades de derecho público y privado dependientes de cualquiera de ellas, así como por las universidades del sistema universitario andaluz. Para el resto de los supuestos tiene el derecho a presentar una reclamación ante la Agencia Española de Protección de Datos.

FINANCIACIÓN

Este estudio no cuenta con fondos específicos actualmente, pero se está concurriendo a convocatorias competitivas autonómicas y nacionales.

RETIRADA DEL CONSENTIMIENTO

Usted puede retirar su consentimiento en cualquier momento sin tener que dar explicaciones. También debe saber que puede ser excluido del estudio si los investigadores del estudio lo consideran oportuno.

Antes de firmar, lea detenidamente el documento, haga todas las preguntas que considere oportunas, y si lo desea, consúltelo con todas las personas que considere necesario. En caso de duda debe dirigirse a la Dra. Adela Maía Gómez González

Firmas:

Firma del paciente:	Firma del Investigador:
Nombre:	Nombre:
Fecha:	Fecha:

*Se deberán firmar dos copias de este documento, una para el participante y otra para la historia clínica.

.
