# Supplementary material for: Effectiveness of adding inspiratory muscle training to a cardiac rehabilitation program in people with acute myocardial infarction revascularized by percutaneous coronary intervention (CARDIOINSPIRE): Study protocol for a randomized controlled trial
Source: PLoS One. 2026 Mar 10;21(3):e0343947. doi: 10.1371/journal.pone.0343947 (PMC12974859; doi:10.1371/journal.pone.0343947)
Supplement: S5 File — (PDF) [file pone.0343947.s005.pdf]

S10 File. CERT Cardiac rehabilitation program and CERT Inspiratory muscle training.

a) CERT Cardiac rehabilitation program.

Section/Topic	Item #	Checklist item	Location **	
			Primary paper (page, table, appendix)	† Other (paper or protocol, website (URL)
**WHAT: materials**	1	Detailed description of the type of exercise equipment (e.g. weights, exercise equipment such as machines, treadmill, bicycle ergometer etc)	Table 1 (page 8 and 9)	
**WHO: provider**	2	Detailed description of the qualifications, teaching/supervising expertise, and/or training undertaken by the exercise instructor	Page 8	
**HOW: delivery**	3	Describe whether exercises are performed individually or in a group	Page 8	
	4	Describe whether exercises are supervised or unsupervised and how they are delivered	Page 8	
	5	Detailed description of how adherence to exercise is measured and reported	Page 8	
	6	Detailed description of motivation strategies	Page 8	
	7a	Detailed description of the decision rule(s) for determining exercise progression	Table 1 (page 8 and 9)	
	7b	Detailed description of how the exercise program was progressed	N/A	
	8	Detailed description of each exercise to enable replication (e.g. photographs, illustrations , video etc)	-	
	9	Detailed description of any home program component (e.g. other exercises, stretching etc)	N/A	
	10	Describe whether there are any non-exercise components (e.g. education, cognitive behavioural therapy, massage etc)	Page 7	

	11	Describe the type and number of adverse events that occur during exercise	N/A
**WHERE: location**	12	Describe the setting in which the exercises are performed	Page 8
**WHEN, HOW MUCH: dosage**	13	Detailed description of the exercise intervention including, but not limited to, number of exercise repetitions/sets/sessions, session duration, intervention/program duration etc	Table 1 (page 8 and 9)
**TAILORING: what, how**	14a	Describe whether the exercises are generic (one size fits all) or tailored to the individual	Table 1 (page 8 and 9)
	14b	Detailed description of how exercises are tailored to the individual	Table 1 (page 8 and 9)
	15	Describe the decision rule for determining the starting level at which people commence an exercise program (such as beginner, intermediate, advanced etc)	N/A
**HOW WELL: planned, actual**	16a	Describe how adherence or compliance to the exercise intervention is assessed/measured	Page 8
	16b	Describe the extent to which the intervention was delivered as planned	N/A

b) CERT Inspiratory muscle training.

Section/Topic	Item #	Checklist item	Location **	
			Primary paper (page, table, appendix)	† Other (paper or protocol, website (URL)
**WHAT: materials**	1	Detailed description of the type of exercise equipment (e.g. weights, exercise equipment such as machines, treadmill, bicycle ergometer etc)	Page 10	
**WHO: provider**	2	Detailed description of the qualifications, teaching/supervising expertise, and/or training undertaken by the exercise instructor	Page 10	
**HOW: delivery**	3	Describe whether exercises are performed individually or in a group	Page 10	

	4	Describe whether exercises are supervised or unsupervised and how they are delivered	Page 10
	5	Detailed description of how adherence to exercise is measured and reported	Page 11
	6	Detailed description of motivation strategies	Page 11
	7a	Detailed description of the decision rule(s) for determining exercise progression	Page 10
	7b	Detailed description of how the exercise program was progressed	N/A
	8	Detailed description of each exercise to enable replication (e.g. photographs, illustrations , video etc)	-
	9	Detailed description of any home program component (e.g. other exercises, stretching etc)	Page 10
	10	Describe whether there are any non-exercise components (e.g. education, cognitive behavioural therapy, massage etc)	N/A
	11	Describe the type and number of adverse events that occur during exercise	N/A
**WHERE: location**	12	Describe the setting in which the exercises are performed	N/A
**WHEN, HOW MUCH: dosage**	13	Detailed description of the exercise intervention including, but not limited to, number of exercise repetitions/sets/sessions, session duration, intervention/program duration etc	Page 10
**TAILORING: what, how**	14a	Describe whether the exercises are generic (one size fits all) or tailored to the individual	Page 10
	14b	Detailed description of how exercises are tailored to the individual	Page 10
	15	Describe the decision rule for determining the starting level at which people commence an exercise program (such as beginner, intermediate, advanced etc)	N/A
**HOW WELL: planned, actual**	16a	Describe how adherence or compliance to the exercise intervention is assessed/measured	Page 10 and 11
	16b	Describe the extent to which the intervention was delivered as planned	N/A
