# Supplementary material for: Effectiveness of adding inspiratory muscle training to a cardiac rehabilitation program in people with acute myocardial infarction revascularized by percutaneous coronary intervention (CARDIOINSPIRE): Study protocol for a randomized controlled trial
Source: PLoS One. 2026 Mar 10;21(3):e0343947. doi: 10.1371/journal.pone.0343947 (PMC12974859; doi:10.1371/journal.pone.0343947)
Supplement: Attachment — Submitted filename: Response to Reviewers.pdf [file pone.0343947.s008.pdf]

Dear Editor,

We are very grateful for the response received after submitting Manuscript PONE-D-25-40583 entitled "Effectiveness of adding inspiratory muscle training to cardiac rehabilitation program in people with acute myocardial infarction revascularized by percutaneous coronary intervention. CARDIOINSPIRE: Study protocol for a randomized controlled trial". The authors have considered the comments made by the reviewers and have integrated the necessary corrections into the manuscript. Undoubtedly, these contributions improved the quality of this study. In the following section, we provide answers to each reviewer's comments.

Sincerely,

Jose M. Zuazagoitia-Lama-Noriega

***RV:** Reviewer

***AA:** Author Answers

<u>REVIEWER #1</u>

RV: Some issues should be added.

RV: 1. A primary end point should be clearly stated and sample size calculation performed on it.

AA: We appreciate this observation and have clearly stated the primary endpoint, which is "Cardiorespiratory Fitness" The sample size calculation was performed according to the meta-analysis by Fabero-Garrido et al. (2024). This study established that people with ischemic heart disease who have undergone an Inspiratory Muscle Training program have a mean difference of 2.18 ml O_2/kg/min with respect to the control group, and we want to find a deviation of 1 MET (3.5 ml O_2/kg/min). The calculations were performed using the statistical software G*Power 3.1, considering a confidence level of 95% and power of 80% in all cases.

- Fabero-Garrido R, Del Corral T, Plaza-Manzano G, Sanz-Ayan P, Izquierdo-García J, López-De-Uralde-Villanueva I. Effects of Respiratory Muscle Training on Exercise Capacity, Quality of Life, and Respiratory and Pulmonary Function in People With Ischemic Heart Disease: Systematic Review and Meta-Analysis. PTJ: Physical Therapy & Rehabilitation Journal | Physical Therapy [Internet]. 2024;

RV: 2. Abstract: in the conclusion I would not put numbers.

AA: Thank you very much for your suggestion. We have reviewed the entire abstract and have only left the number corresponding to the Clinical Trials registry, as this is a requirement of the journal's guidelines.

RV: 3. Methods: probably more details about rehabilitation should be added in the appendix.

AA: We have incorporated the Consensus on Exercise Reporting Template (CERT) as supporting information (S10 File) to include all the details of the rehabilitation and ensure that the exercise intervention can be replicated.

RV: 4. Methods: ways of blinding should be better detailed.

AA: In a specific section entitled "Blinding" (pages 19-20, lines 383-395), we have adequately detailed the blinding of all parties involved: participants, rehabilitation physicians, evaluators, physical therapist and nurse who will perform the intervention, and statistician who will analyze the data. The following text was added to the main document:

"Participants will receive instruction about IMT from one of the rehabilitation physicians, who will be different from the one performing the assessments. Furthermore, none of the participants will know to which group they belong, since all of them will be given the same IMT device, but with a different training load.

The physiotherapist and nurse who will conduct the CRP training sessions will not be involved in the allocation of participants.

The statistical analysis will be carried out by a statistician of the research team who will not participate in the allocation, assessment or intervention process. In addition, the participants' data will be provided anonymously.

In the event of any adverse effect or circumstance that endangers the health of the participants, the researchers will be unblinded. If such a contingency arises, both a paper-based and an electronic record are always maintained and remain accessible to all authorized investigators."

RV: 5. Methods: it should be added if block sizes were exploited.

AA: We thank the reviewer for this important observation. We have revised and significantly expanded the description of the random sequence generation process (page 18, lines 368-381). Each patient will be randomly assigned to either the intervention or control group (1:1 ratio) using a random sequence of numbers generated with the Simple Random Sampling module of Epidat 3.1 (open-access epidemiological analysis software developed by the Xunta de Galicia, Dirección Xeral de Saúde Pública). Specifically, to reach a sample size of N = 72, the software will generate random numbers that will identify the participants. Participants will be randomly assigned to the intervention group or control group based on the list of random numbers generated by the software. Although block sizes will not be exploited, this simple random assignment will ensure maximum unpredictability in the assignment of participants, preventing any prediction of future assignments.

REVIEWER #2

Major Comments

RV: 1. Major Comment — Eligibility Criteria and Terminology ("Myocardial Ischemia"). Page 6, line 121:

The term "myocardial ischemia (MI)" is potentially ambiguous and requires clearer definition. It is not evident whether the authors refer to individuals with acute myocardial infarction or to those with conditions involving reduced myocardial perfusion (e.g., stable coronary artery disease). Please specify the objective inclusion criteria (e.g., diagnostic biomarkers, ECG findings, imaging evidence) to ensure accurate characterization of the study population. Additionally, clarify whether the sample includes participants undergoing elective procedures, emergency procedures, or both. If a more precise descriptor is warranted, please apply the updated terminology consistently throughout the manuscript, including the title, abstract, introduction, and the "Participants and Study Setting" section.

AA: We greatly appreciate your feedback. We have replaced the term "myocardial ischemia" with "acute myocardial infarction" (code: BA41 from the International Classification of Diseases, 11th revision), which specifically defines the study population and, therefore, as you suggest, is more accurate. In addition, we have added as an inclusion criterion (page 6, line 124) that an emergency percutaneous coronary intervention has been performed. With these changes, it is clear that people with stable angina or those who have undergone a scheduled procedure will not be included. We have also updated all sections of the manuscript accordingly.

RV: 2. Major Comment — Terminology: "Functional Capacity" vs. "Cardiorespiratory Fitness"

Page 9, lines 169–170: In the Outcomes section, the term "functional capacity" is used to describe VO_2max obtained during maximal exercise testing. These measures are more accurately characterized as indicators of cardiorespiratory fitness rather than functional capacity in the clinical sense. I recommend replacing "functional capacity"

with “cardiorespiratory fitness” in this section and ensuring that any earlier or subsequent uses of the term are revised for consistency and conceptual clarity.

AA: Thank you very much for your comment. Indeed, the term “cardiorespiratory fitness” is more consistent, so we have replaced “functional capacity” with “cardiorespiratory fitness”. We have also revised the term throughout the rest of the document.

RV: 3. Major Comment — Clarification of Revascularization Terminology (PTCA vs. PCI) Page 6, line 109.

The terminology used to describe the revascularization procedure (“percutaneous transluminal coronary angioplasty – PTCA”) appears potentially inaccurate or incomplete. Contemporary cardiology literature typically uses the broader term percutaneous coronary intervention (PCI), which encompasses both balloon angioplasty (PTCA) and stent implantation. Because the manuscript title and several sections refer specifically to PTCA, it remains unclear whether the study will include only patients treated with balloon angioplasty, those treated with PCI involving stents, or both types of coronary interventions. This distinction should be clearly specified in the Eligibility Criteria section.

If the intention is to include all forms of PCI, the terminology should be updated consistently throughout the manuscript, including the title, abstract, introduction, and the Participants and Study Setting section, as well as all subsequent occurrences. Conversely, if the study is designed to include exclusively patients treated with PTCA, this should be explicitly stated and adequately justified, given that isolated PTCA is less common in contemporary clinical practice.

AA: We appreciate your suggestion and agree with it. We have changed the term “percutaneous transluminal coronary angioplasty (PTCA)” to “percutaneous coronary intervention (PCI)”. This clarifies the group of people we want to study which encompasses both balloon angioplasty (PTCA) and stent implantation. The terminology used in the manuscript in this regard has been modified accordingly.

RV: 4. Major Comment — Adoption of the CONSORT, SPIRIT, and CERT Frameworks

In addition to the SPIRIT checklist for study protocols and the CONSORT guidelines for the forthcoming randomized clinical trial, I recommend incorporating the Consensus on Exercise Reporting Template (CERT) (doi:10.1136/bjsports-2016-096651). CERT will help ensure comprehensive reporting of exercise-related components in both the protocol and the final trial manuscript.

AA: We have incorporated the Consensus on Exercise Reporting Template (CERT) as Supporting Information (S10 File) to ensure comprehensive reporting of exercise-related components.

RV: 5. Major Comment — Allocation Concealment Not Adequately Ensured

The description of the allocation process suggests that group assignment may be predictable, which raises concerns regarding allocation concealment. Concealment must be ensured so that neither participants nor investigators involved in enrollment can foresee upcoming assignments. Please clarify the allocation procedure and describe the mechanism used to maintain adequate concealment (e.g., central randomization, sealed opaque envelopes). Notably, allocation concealment is conceptually distinct from blinding and must be addressed separately. This concern arises from the paragraph describing group allocation.

AA: We greatly appreciate your comments. We have rewritten the “Allocation Concealment” section (pages 18-19, lines 367-381) and separated it from the “Blinding” section (pages 19-20, lines 383-395) as you requested. We agree that a robust allocation concealment mechanism is fundamental to preventing selection bias. To ensure that the assignment is unpredictable and independent of the investigators involved in recruitment, we will implement the following actions: a) The randomization sequence will be generated by a team member who will not be involved in the clinical recruitment or assessment of participants, using Epidat 3.1 software. b) This independent researcher

will prepare a set of identical, opaque, and carbon-lined envelopes. Each envelope will be sequentially numbered on the outside and it will contain the group assignment on a folded card inside. c) The envelopes will be opened by the rehabilitation physician only after the patient meets all eligibility criteria and provides written informed consent. The envelopes will be opened in strict numerical order, ensuring that the assignment for the next participant will remain unknown until the previous one is fully enrolled.

RV: 6. Major Comment — Clarity in Random Sequence Generation.

The manuscript does not provide sufficient detail on how the random allocation sequence will be generated. Although Epidat 3.1 is mentioned, the specific method used (e.g., computer-generated random numbers, block randomization, stratification) is not described. Please specify how the software generates the random sequence, including any parameters used.

AA: Thank you very much again for the improvements you requested. Accordingly, the following text was added (page 18, lines 370-377): *"Once included, each patient will be randomly assigned to either the intervention or control group (1:1 ratio) using a random sequence of numbers generated with the Simple Random Sampling module of Epidat 3.1 (open-access epidemiological analysis software developed by the Xunta de Galicia, Dirección Xeral de Saúde Pública). Specifically, to reach a sample size of n = 72, the software will generate a sequence that determines the group for each participant according to their order of enrollment. This simple random assignment will ensure maximum unpredictability in the assignment of participants, preventing any prediction of future assignments."*

RV: 7. Major Comment — Statistical Analysis

The current statistical plan relies on multiple independent tests for within- and between-group comparisons. For a randomized trial with pre–post measurements, this approach does not adequately evaluate the group × time interaction. I recommend specifying a unified model (e.g., repeated-measures ANOVA or a linear mixed-effects model) to

assess differential changes between groups over time. Additionally, the authors should explicitly state that analyses will follow the intent-to-treat principle and that the significance level will be set at 5% with 95% confidence intervals.

AA: Thank you for your valuable comment. We have specified that the data will be analyzed using a mixed-design ANOVA to assess differential changes between groups over time. And we have also established that an intention-to-treat analysis with multiple imputation will be implemented for missing data exceeding 5%; otherwise, a complete case analysis will be conducted. Furthermore, we have specified that the significance level will be set at 5% with 95% confidence intervals.

Minor Comments — Clarifications and Reference Updates

RV: 8. Page 4, line 79: Endurance and strength training are mentioned, but no supporting reference is provided. Please include an appropriate citation.

AA: We have included the following reference that clearly highlights the obligation to include strength training and aerobic exercise in cardiac rehabilitation programs.

- Brown TM, Pack QR, Aberegg E, Brewer LC, Ford YR, Forman DE, et al. Core Components of Cardiac Rehabilitation Programs: 2024 Update: A Scientific Statement From the American Heart Association and the American Association of Cardiovascular and Pulmonary Rehabilitation. Circulation [Internet]. 2024 Oct 29;150(18):e328–47.

RV: 9. Page 5, line 88: The citation of the study by Fabero-Garrido may give the impression that the sample included only individuals undergoing PTCA. However, the original study included optimally treated individuals with ischemic heart disease, regardless of revascularization status. Please revise to ensure accurate representation.

AA: Thank you very much for your comment. We have rewritten that sentence (page 5, line 87), specifying that Fabero-Garrido's study includes patients with ischemic heart disease so as not to give the impression that it only includes patients undergoing PTCA.

RV: 10. Page 5, line 94: The abbreviation "MIP" appears for the first time. Please define it as "maximal inspiratory pressure (MIP)" at first mention.

AA: We appreciate you pointing that out, and we have defined "maximal inspiratory pressure" for the first time.

Minor Comment — Specification of Exercise Contraindications

RV: Pages 6–7, lines 126–127: The manuscript states that contraindications to exercise will be considered but does not list them. Please provide the specific contraindications or cite the guideline used to define them.

AA: Thank you for your insightful comment. We have added the main specific contraindications for starting cardiac rehabilitation in patients who have suffered an acute myocardial infarction and have added the corresponding bibliographic reference:

- Protoview P, Vol B, Ringgold B. Guidelines for Cardiac Rehabilitation Programs, 6th Edition. ProtoView [Internet]. 2020 [cited 2025 Dec 19];2020(19):1–2.

Minor Comment — Justification of the IMT Protocol Intensity

RV: Page 8, lines 155–157: The inspiratory muscle training protocol (70% of MIP, 3 sets of 10 repetitions performing one daily training session four days a week) requires clearer justification. This intensity appears relatively high, and it is not clear whether this configuration has been tested in similar populations or shown to be effective and well tolerated. If supported by previous evidence, please cite the relevant studies. Otherwise, consider whether a more gradual progression of the training load—particularly during the initial sessions—might improve tolerance and adherence.

AA: The next three studies, which involved coronary artery bypass graft surgery and coronary artery disease patients and used fairly similar IMT protocols in terms of sets, repetitions, weekly sessions, and intensities (up to 80% of MIP), had no adverse effects, supporting the feasibility and safety of high-intensity IMT in this population. For this reason, we selected 70% MIP as a high-intensity stimulus, maintaining a conservative margin below the highest tested intensity (80% MIP) to balance effectiveness and tolerability. The first study found benefits in exercise capacity, inspiratory muscle strength, quality of life, and antioxidant profile. The second trial showed improvement in the apnea-hypopnea index. The third trial reported no significant between-group differences in the main outcomes.

1. dos Santos TD, Pereira SN, Portela LOC, Cardoso DM, Lago PD, dos Santos Guarda N, et al. Moderate-to-high intensity inspiratory muscle training improves the effects of combined training on exercise capacity in patients after coronary artery bypass graft surgery: A randomized clinical trial. Int J Cardiol. 2019 Mar 15;279:40–6.
2. Labeix P, Berger M, Zellag A, Garcin A, Barthelemy JC, Roche F, et al. Resistance Training of Inspiratory Muscles After Coronary Artery Disease May Improve Obstructive Sleep Apnea in Outpatient Cardiac Rehabilitation: RICAOS Study. Front Physiol. 2022 Mar 3;13.
3. Paula Miozzo A, Stein C, Zago Marcolino M, Rebolho Sisto I, Hauck M, Corrêa Coronel C, et al. Effects of High-Intensity Inspiratory Muscle Training Associated with Aerobic Exercise in Patients Undergoing CABG: Randomized Clinical Trial. Brazilian Journal of Cardiovascular Surgery Braz J Cardiovasc Surg. 2018;33(4):376–83.

Minor Comment — Appropriateness of the NYHA Scale

RV: Page 15, line 305: It is not clear whether all participants will have a diagnosis of heart failure. If not, the NYHA classification may not be the most appropriate tool for assessing dyspnea, as it is designed specifically for heart failure populations. For a broader cardiac sample, I recommend using an alternative validated instrument, such as the modified Medical Research Council scale (mMRC), which is applicable across

different etiologies of dyspnea and may provide a more suitable assessment for this study.

AA: We appreciate the reviewer's comment. Although NYHA was originally developed for heart failure, it is widely used in clinical cardiology and cardiac rehabilitation to classify functional limitation, particularly exertional symptoms, across cardiac conditions. Given that it is routinely used in our setting and will be applied by trained clinicians using standardized criteria, we will retain NYHA for consistency and clinical interpretability.
